# Polyandry: A threat or an opportunity for the sterile insect technique?

**DOI:** 10.1371/journal.pcbi.1014212

**Published:** 2026-04-29

**Authors:** Marine A. Courtois, Louise van Oudenhove, Suzanne Touzeau, Frédéric Grognard, Ludovic Mailleret

**Affiliations:** 1 Université Côte d’Azur, INRAE, ISA, France; 2 Université Côte d’Azur, Inria, INRAE, CNRS, MACBES, France; University of Cambridge, UNITED KINGDOM OF GREAT BRITAIN AND NORTHERN IRELAND

## Abstract

The sterile insect technique (SIT) is a pest control strategy based on the mass release of sterilized males to disrupt natural reproduction and suppress wild populations. However, its effectiveness can be challenged by biological factors such as female multiple mating and sperm use bias. While multiple mating is widespread among many insect species, the mechanisms governing sperm use remain poorly understood. In this study, we develop and analyze a compartmental mathematical model based on differential equations to investigate the overall impact of multiple mating on SIT efficiency. We further analyze the effect of sperm use biases with an agent-based model, calibrated on *Drosophila suzukii*, allowing the exploration of different scenarios: preferential use of first vs last sperm, of fertile vs sterile sperm, and mixed sperm use. Our results highlight how multiple mating and sperm use biases influence SIT effectiveness. In the longer term, multiple mating is disadvantageous as it requires additional releases of sterilized males to control the pest population. However, in the shorter term, it can be beneficial by disrupting further female reproductive output by “defertilizing” females mated with wild males. This study provides new information on how the way sperm is processed after mating can impact sterile insect control strategies, highlighting the limited influence of these biological processes depending on the release efforts that can be deployed.

## Author summary

Insect pests are a major threat to agriculture worldwide. One environmentally friendly method to control their populations is the sterile insect technique (SIT), which consists of releasing sterilized males into the wild. These males cannot produce offspring, so if females mate only once, the population declines. However, in many insect species, females can mate several times with different males, and they do not always use sperm randomly. In this study, we used mathematical and computer models to better understand how such behaviors influence the success of SIT. We first built a simplified model to study the general impact of multiple mating. Then, we created a more detailed simulation based on the biology of *Drosophila suzukii*, a highly damaging fruit pest. This simulation allowed us to test what happens when females prefer to use sperm from certain males over others. Our results show that multiple mating can sometimes help control the population in the short term, but makes long-term suppression more difficult. This work helps improve our understanding of how complex mating behaviors affect pest control strategies and may contribute to the design of release programs.

## 1 Introduction

The sterile insect technique (SIT) is an autocidal pest control method: it uses the pest species to control its own population by regular inundative releases of sterilized males [1]. Insects of the target species are mass-reared, sexed (when possible), and then sterilized before being released in large numbers. Once released, sterilized males compete with wild males for mates, reducing the proportion of females that produce viable offspring. This leads progressively to a decline of the pest population size. Originally conceptualized by the entomologist E. F. Knipling in the 1950s [2], SIT was first used to eradicate the New World screwworm fly (*Cochliomyia hominivorax*) [3]. Today, SIT offers a sustainable alternative or complementary approach to chemical control methods in various pest management programs (Table 1). Regarding human health, SIT has been applied to control disease vectors such as mosquitoes [1]. In agriculture, it has been used against pests such as the melon fly *Bactrocera cucurbitae* and the Mediterranean fruit fly *Ceratitis capitata* [55]. Recently, SIT programs have been extended to new invasive pests, including *Drosophila suzukii*, commonly known as the Spotted Wing Drosophila (SWD). *Drosophila suzukii* is one of the most damaging pests in soft fruit crops, particularly affecting berries, cherries, and sometimes grapes and peaches [56,57]. Unlike other *Drosophila* species, *D. suzukii* targets healthy, non-rotting fruits, causing severe economic losses due to larval feeding and oviposition wounds, which favour pathogen introduction [58,59]. Due to its broad host range and aggressive damaging of healthy fruits, *D. suzukii* represents a major agricultural challenge worldwide. Current control strategies heavily rely on chemical methods, which poses environmental and resistance-related challenges, thereby encouraging the exploration of alternative approaches such as SIT [59].

**Table 1:** Summary of species targeted by sterile insect technique (SIT) programs: Location represents where SIT has been implemented; Polyandry? indicates whether the species is polyandrous (multiple matings) or not (single mating per female); Date indicates the date or approximate time frame for the initial study on the implementation of SIT for the species; Reference cites key studies.

Species	Location	Polyandry?	Date	Reference
**Livestock pests**				
*Cochliomyia hominivorax* (New World screwworm)	USA, Mexico, Central America, Libya	No	1950s	[4,5]
*Chrysomya bezziana* (Old World screwworm)	Papua New Guinea, Australia	No	1989	[6,7]
**Agricultural pests: Fruit flies (Tephritidae)**				
*Bactrocera dorsalis* (Oriental fruit fly)	Thailand, China, East Africa	Yes	1960s	[1,8]
*Ceratitis capitata* (Mediterranean fruit fly)	Worldwide (Spain, Croatia, Argentina, Brazil, Israel etc.)	Yes	1970s	[1,9]
*Anastrepha ludens* (Mexican fruit fly)	Mexico, USA	Yes	1980s	[10]
*Anastrepha obliqua* (West Indian fruit fly)	Mexico, Central America	Yes	1980s	[11]
*Bactrocera tryoni* (Queensland fruit fly)	Australia	Yes	1980s	[12]
*Zeugodacus cucurbitae* (Melon fruit fly)	USA, Japon, Inde, Africa	Yes	1980s	[13]
*Bactrocera oleae* (Olive fruit fly)	Mediterranean region, North America, Israel	Yes	2000s	[1,14]
**Agricultural pests: Other insects**				
*Pectinophora gossypiella* (Pink bollworm)	Mexico, USA	Yes	1960s	[15,16]
*Eldana saccharina* (African sugarcane borer)	South Africa	No	1970s	[17,18]
*Lymantria dispar* (Gypsy moth)	USA	No	1975	[15,19]
*Delia antiqua* (Onion maggot)	USA, Europe, Israel, Netherlands	No	1981	[1,20]
*Anthonomus grandis* (Boll weevil)	Mexico, Brazil, USA	Yes	1990s	[21,22]
*Cactoblastis cactorum* (Cactus moth)	USA, Mexico	Yes	1990s	[23]
*Cydia pomonella* (Codling moth)	Canada	No	1990s	[1,24]
*Cylas formicarius* (Sweetpotato weevil)	Japan	Yes	2000s	[25,26]
*Ectomyelois ceratoniae* (Carob moth)	Tunisia, Iran	Yes	2000s	[27]
*Euscepes postfasciatus* (Sweetpotato weevil)	Japan	Yes	2000s	[28,29]
*Helicoverpa armigera* (Cotton bollworm)	China	Yes	2000s	[30,31]
*Lobesia botrana* (European grapevine moth)	Chile	No	2000s	[15,32]
*Liriomyza trifolii* (Leafminer)	USA	Yes	2000s	[33–35]
*Thaumatotibia leucotreta* (False codling moth)	South Africa	Yes	2000s	[36,37]
*Aethina tumida* (Small hive beetle)	USA, Australia	Yes	2010s	[38,39]
*Amyelois transitella* (Navel orangeworm)	USA	No	2010s	[40,41]
*Halyomorpha halys* (Brown marmorated stink bug)	USA, Europe	Yes	2010s	[42,43]
*Drosophila suzukii* (Spotted-wing drosophila)	Europe, North America	Yes	2020s	[44,45]
*Spodoptera frugiperda* (Fall armyworm)	Americas, Africa, Asia	Yes	2020s	[46]
**Disease vectors**				
*Aedes aegypti* (Dengue mosquito)	Brazil, Thailand, China, USA, Australia, Mexico, Europe, Mauritius	Yes	1960s	[47,48]
*Glossina spp.* (Tsetse fly)	Sub-Saharan Africa, Burkina Faso	No	1970s	[1,49,50]
*Anopheles arabiensis* (Malaria mosquito)	Sub-Saharan Africa	Yes	2000s	[51,52]
*Aedes albopictus* (Asian tiger mosquito)	Europe, Asia, Americas	Yes	2010s	[53,54]

The successful implementation of SIT depends on identifying key biological and technical factors that influence its outcomes. Among these, the mating behavior of the target species, particularly polyandry, has been highlighted as an influential aspect. Historically, SIT programs have targeted species where females are predominantly monogamous, i.e., monoandrous, mating only once in their lifetime [2]. Knipling (1955) proposed that one principle to consider when determining if SIT is appropriate for a species was: “Females must normally mate only once.” [2]. However, increasing attention has been given to polyandrous species, in which females mate with multiple males over their reproductive lifetime. The timing and frequency of such matings are often constrained by refractory rates (i.e., intervals between matings). In such species (Table 1), multiple mating can significantly shape population dynamics because females mating with multiple males may reduce the chances that sterilized males successfully prevent reproduction. Various techniques have been developed to mitigate re-mating in polyandrous species, such as dietary modifications or semiochemical treatments that enhance the ability of sterilized males to suppress further mating [60,61]. For example, in *Ceratitis capitata*, a polyandrous species, sterilized males treated with ginger root oil inhibit female re-mating more effectively than untreated males [62,63].

While polyandry could be perceived as a challenge for SIT, its impact may largely depend on factors such as mating order, sperm storage, and sperm competition. Early theoretical studies reconsidered the necessity of monogamous mating systems for SIT success [3,64], emphasizing that the key determinant is the competition between wild and sterile sperm, rather than the number of matings per female [65]. More recent works support this view, showing that as long as sterilized male sperm remains competitive and mating occurs randomly, polyandry is not inherently incompatible with SIT, although its effectiveness can be compromised when wild male sperm is preferentially used by females for fertilization [1]. The impact of re-mating on SIT efficiency hence depends on sperm selection biases, among other factors such as male fertility influence on re-mating probability [66,67]. These processes can either dilute or bypass the sterilizing effect of the initial mating.

In the context of *D. suzukii*, multiple mating behavior and sperm use biases are particularly relevant. The females of this species can mate with multiple males, being able to produce offspring from up to five mates [68]. Additionally, SWD females possess two spermathecae, which may enable differential sperm storage and use [69]. The mechanisms driving sperm selection and use in SWD remain poorly understood, studies on related species provide valuable insights. For instance, in *Drosophila melanogaster*, sperm storage structures can influence sperm use: some genotypes are associated with a preferential use of first-mating sperm due to anatomical features limiting storage of later sperm [70], while other studies report last-male precedence, with the most recent male siring over 80% of offspring [71]. These contrasting findings highlight the complexity of post-copulatory processes and sperm use. Reflecting this complexity, mixed sperm use strategies, where females store and use sperm from multiple males simultaneously, have also been proposed [72]. Sterilization processes may further influence SIT outcomes by reducing sperm quantity or altering ejaculate composition. For example, irradiated mosquitoes produce significantly fewer sperm than un-irradiated males [73]. While no equivalent data exist for SWD, lower sperm numbers in sterilized males could reduce the likelihood of successful sperm use. Similar effects have been observed in other insect species (such as corn earworm and boll weevil), where sterilization-induced sperm reduction can lower sterilized male competitiveness by promoting female re-mating with wild males [74,75]. Additionally, studies suggest that mating duration correlates positively with male ejaculate investment [76], and that longer copulations increase the likelihood of that sperm being used in polyandrous contexts [77]. Further, mating plugs, such as those observed in *Drosophila hibisci*, may limit the access of subsequent males’ sperm to the female reproductive tract, thereby favoring first-mating sperm [78].

This study aims to evaluate the impact of multiple mating, in particular sperm use biases on SIT outcomes using mathematical modeling to explore different scenarios and hypotheses. Various modeling studies have already contributed to advance knowledge on SIT effectiveness by investigating key factors such as release strategies [79], residual fertility in sterilized males released [9,80–83], sterilized male competitiveness [84] or female mate preferences [85,86]. Interestingly, although re-mating is generally considered detrimental to SIT efficiency, modeling studies focusing on the specific case of two successive matings showed that in certain cases, depending on female reproductive parameters, it may enhance SIT outcomes [9,83]. More generally, SIT models incorporating multiple mating have either relied on a single-remating structure [9,83] or considered multiple mating events in a more generic framework [87,88], without explicitly disentangling the consequences of alternative sperm-use strategies.

We adopt a multi-model approach to study the influence of polyandry and sperm-use biases on pest population dynamics under SIT. We first develop a compartmental model based on ordinary differential equations, which provides a global understanding of how re-mating affects qualitative results and population-level outcomes. We then complement this with an agent-based model (ABM), calibrated on SWD, that enables us to investigate more detailed scenarios of sperm use associated with multiple mating events. In particular, we explore various sperm use strategies, including preferential use of first sperm, preferential use of last sperm, mixed sperm use, and female preference for fertile or sterile sperm. Previous studies have used ABMs to explore SIT outcomes, such as eradication timelines [89], small-scale spatial effects [90], or enhanced intervention strategies [91]. Here, we use the agent-based framework specifically to investigate how sperm-use biases affect SIT efficiency. While the modelling framework remains general, the agent-based simulations are conducted as a case study parameterised on a realistic commercial strawberry tunnel scenario, one of the main cropping systems affected by SWD, with population dynamics observed over a 100-day production period. This allows us to evaluate how sperm-use biases would impact SIT efficiency in field-like conditions. This dual modeling framework helps evaluate the robustness of SIT under different biological assumptions and sheds light on the role of female post-mating processes in shaping control outcomes.

This article is structured as follows: Section 2.1 presents the compartmental model, which explores the global dynamics of re-mating. Section 2.2 introduces the agent-based model for analyzing finer-scale sperm use biases and their implications for SIT. Section 3 presents in details the results obtained for both models. Finally, Section 4 discusses the impact of re-mating and sperm use biases on population dynamics, the complementarity of the two approaches, their implications for optimizing SIT, and perspectives for further refining this pest control method.

## 2 Methods

### 2.1 Compartmental modeling: Impact of multiple mating on SIT effectiveness

Several mathematical models have incorporated multiple mating in the context of SIT. The models proposed in [83] and [9] are based on a single-remating structure. More specifically the model in [9] is extended in [83] through the addition of an immature stage that make it possible to account for larval stage-specific mortality. In contrast, the models in [87,88] consider multiple mating events, the former in a generic framework and the latter extending it by including the effect of sterilized males. From a biological standpoint, the latter approach is particularly relevant for species such as *Drosophila suzukii*, where females frequently remate: a minimum of 2 and a maximum of 5 matings per female were reported in [92] (2.68 + /-1.14 in average) and these numbers can even reach higher values according to [68] (3.25 + /-0.46 in average). Consequently, while models assuming a single re-mating (e.g., [9,83]) could be considered for *D. suzukii* under fairly conservative assumptions, we adopt here a more general behavioural framework, building on [88]’s modelling framework.

#### 2.1.1 Model description.

**Fig 1:**
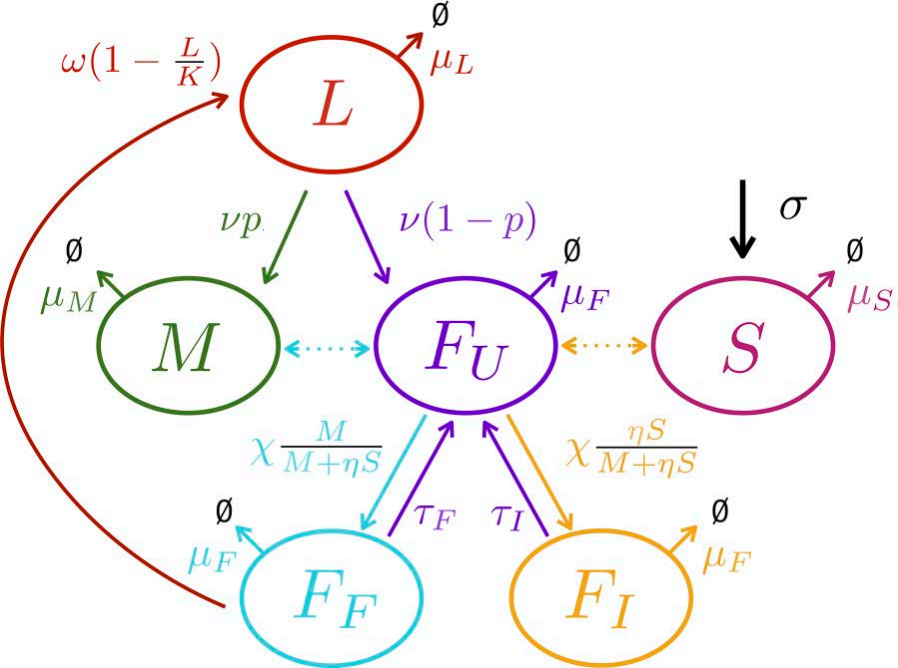
Flow diagram of the population dynamics model (Eq 1). The compartments (and color code) correspond to larvae *L* (red), wild males *M* (green), sterilized males *S* (pink), unmated females *F*_*U*_ (purple), fertilized females *F*_*F*_ (blue) and infertile females *F*_*I*_ (orange). Solid arrows correspond to flows, dotted arrows to matings. Sterilized males are released at rate σ. Reproduction is represented by the egg-laying rate ω, the larvae hatching rate ν, the proportion of males among offspring *p* and the proportion of successfull matings $\frac{M}{M+\eta S}$. Finally, all compartments are affected by specific mortality rates μ.

The model describes the population dynamics of the following compartments: immature stage *L* (pre-adults including all immature sub-stages: eggs, larvae, and pupae, hereafter referred to as ’larvae’), wild males *M*, sexually mature females that are yet unmated or available for re-mating *F*_*U*_, infertile females *F*_*I*_ (females mated with sterilized males), fertilized females *F*_*F*_ (females mated with wild males), and sterilized males *S* released as part of the sterile insect technique deployment (Fig 1). The system is defined as follows:


$$\left\{ \begin{array}{c} \dot{L}=\omega (1-\frac{L}{K})F_{F}-(\mu_{L}+\nu )L,\\ \dot{M}=\nu pL-\mu_{M}M,\\ \dot{F_{U}}=\nu (1-p)L+\tau_{F}F_{F}+\tau_{I}F_{I}-(\mu_{F}+\chi )F_{U},\\ \dot{F_{I}}=\chi \frac{\eta S}{M+\eta S}F_{U}-(\tau_{I}+\mu_{F})F_{I},\\ \dot{F_{F}}=\chi \frac{M}{M+\eta S}F_{U}-(\tau_{F}+\mu_{F})F_{F},\\ \dot{S}=-\mu_{S}S+\sigma . \end{array}\right.$$ (1)

with the dot notation representing the time derivative.

The dynamics of larvae *L* are affected by their mortality $\mu_{L}$, the larval hatching rate ν, and the egg-laying rate of fertilized females (*F*_*F*_) ω. Larval competition is represented by a logistic-like competition function through the load-carrying capacity *K*, i.e., the maximum number of juveniles that can be contained in the fruits. Resources for oviposition and larval development are assumed to be finite and constant. The dynamics of males *M* are driven by their mortality $\mu_{M}$ and the emergence of new males, which is dependent on the larval hatching rate ν and sex ratio *p*. The dynamics of unmated and available females *F*_*U*_ depend on their mortality $\mu_{F}$, the emergence of unmated females (with larval hatching rate ν and sex ratio *p*) and the rate at which females mate χ. The dynamics of *F*_*U*_ are also influenced by females that become available again, with a refractory rate $\tau_{F}$ for fertilized females *F*_*F*_ and a refractory rate $\tau_{I}$ for infertile females. The refractory rate is defined as the inverse of time required for a mated female to become available for mating again. The dynamics of fertilized females *F*_*F*_ (respectively infertile females *F*_*I*_) are affected by their mortality $\mu_{F}$, the proportion of unmated females *F*_*U*_ that mate with a wild male *M* (respectively a sterilized male *S*) and the refractory rate $\tau_{F}$ (respectively $\tau_{I}$) of females that become available again. We assume here that males are not limiting so that successful matings only depends on the ratio between fertile males and attractive sterilized males, with parameter η accounting for sterilized males competitiveness. Finally, the dynamics of sterilized males *S* are affected by their mortality $\mu_{S}$ and the release rate σ.

The parameter values used, calibrated for *Drosophila suzukii*, are listed in Table 2. Details on the extraction and calculation of parameter values are provided in S1 Text. As said in the introduction, *D. suzukii* is known to be polyandrous ([68,76,96], Table 1). Male availability is not a limiting factor, nor is their mating capacity. Wild and irradiated males of this species have been reported to possibly achieve more than six matings within a 24-hour period [76]. Females of the species *D. suzukii* typically mate shortly after their emergence, once they reach sexual maturity [97]. Moreover, the high density of males promotes rapid interactions and immediate mating.

**Table 2:** Model parameters estimated for *Drosophila suzukii.*

Parameter (Description)	Value	Unit	Reference
$\mu_{L}$ (Larvae mortality rate)	0.037	day^−1^	[93]
$\mu_{F}$ (Female mortality rate)	0.012	day^−1^	[94]
$\mu_{M}$ (Male mortality rate)	0.013	day^−1^	[94]
$\mu_{S}$ (Sterilized male mortality rate)	0.054	day^−1^	[76]
ω (Egg-laying rate)	6	eggs per female per day	[93]
ν (Larvae hatching rate)	0.08	day^−1^	[93]
*p* (Sex ratio)	0.50	–	[93]
χ (Mating rate)	10	day^−1^	[95]
η (Sterilized male competitiveness)	0.60	–	[95]
*K* (Carrying capacity)	36,000	larvae per tunnel	Calculated^1^
σ (Sterilized male release rate)	–	ind.day^−1^	–
$\tau_{F}$ (Refractory rate of fertilized females)	0.12	day^−1^	[96]
$\tau_{I}$ (Refractory rate of infertile females)	0.14	day^−1^	[96]

^1^Estimation based on larval density calculations from *D. suzukii* for a strawberry tunnel of approximately 500 *m*^2^ (more details in S1 Text).

Therefore, we assume in our model that females remain in the unmated compartment (*F*_*U*_) for a very short time, reflecting this pest’s ability to quickly colonize environments through efficient reproduction [98]. Indeed, the mating rate χ is of the order of 10 per day, this is roughly 100 times larger than ν and about 1000 times larger than the mortality rates (Table 2). Based on this assumption, we perform a slow-fast approximation, as described in S2 Text. In this case, *F*_*U*_ rapidly tends to zero. This approximation allows us to reduce the system (Eq 1) to the following model without *F*_*U*_:


$$\left\{ \begin{array}{c} \dot{L}=\omega \left(1-\frac{L}{K}\right)F_{F}-(\mu_{L}+\nu )L,\\ \dot{M}=\nu pL-\mu_{M}M,\\ \dot{F_{I}}=\nu (1-p)\frac{\eta S}{M+\eta S}L+\tau_{F}\frac{\eta S}{M+\eta S}F_{F}-(\mu_{F}+\tau_{I}\frac{M}{M+\eta S})F_{I},\\ \dot{F_{F}}=\nu (1-p)\frac{M}{M+\eta S}L+\tau_{I}\frac{M}{M+\eta S}F_{I}-(\mu_{F}+\tau_{F}\frac{\eta S}{M+\eta S})F_{F},\\ \dot{S}=-\mu_{S}S+\sigma . \end{array}\right.$$ (2)

#### 2.1.2 Analysis of equilibria.

The dynamics of the sterilized males *S* are decoupled from the other state variables, and *S* tends to its equilibrium value $S^{*}=\frac{\sigma }{\mu_{S}}$. Equilibrium values for other variables are obtained for *S* = S^*^ (see S4 text). There are between one and three equilibria for the model (Eq 2). Among these equilibria, the pest-free equilibrium, denoted as $E_{0}^{*}$, always exists and represents a case where the pest population is completely eradicated. This pest-free equilibrium $E_{0}^{*}$ is shown to be locally asymptotically stable as soon as σ>0, as demonstrated in S3 Text.

In addition to this, infestation equilibria, which correspond to cases where the pest population persists, are obtained from the solutions of the equation $\zeta (L^{*})=\sigma$, where L^*^ denotes the larval population at equilibrium (S4 Text):


$$\zeta (L^{*})=\frac{\gamma \mu_{S}(\mu_{F}+\tau_{I})}{\eta (\mu_{F}+\tau_{F})}\left(-\frac{{ℛ}_{0}}{K}L^{*2}+({ℛ}_{0}-1)L^{*}\right),\;\text{with }\gamma =\frac{\nu p}{\mu_{M}}.$$ (3)

In this expression, the basic reproduction number, is defined as: ${ℛ}_{0}=\frac{\omega (1-p)\nu }{\mu_{F}(\mu_{L}+\nu )},$ and represents the average number of viable female offspring produced by a single pest female.

The parabola described by $\zeta (L^{*})$ allows us to identify the conditions that lead to pest population eradication or persistence by analyzing how larval density at equilibrium L^*^ responds to variations in the release intensity σ. In order to determine the stability of this infestation equilibria, a numerical analysis was conducted on the eigenvalues of the Jacobian matrix associated with the system at these equilibria (S4 Text).

We define the tipping point $\bar{\sigma }$ as the critical release rate of sterilized males beyond which the pest-free equilibrium becomes the only stable state of the system. This threshold, referred to as the *eradication threshold*, corresponds to the minimum number of sterilized males σ that must be released to ensure effective control of pest populations in agricultural systems. Mathematically, it corresponds to the summit of parabola (Eq 3), and is thus given by:


$$\bar{\sigma }=\frac{K\gamma \mu_{S}({ℛ}_{0}-1{)}^{2}(\mu_{F}+\tau_{I})}{4\eta {ℛ}_{0}(\mu_{F}+\tau_{F})}.$$ (4)

**Fig 2:**
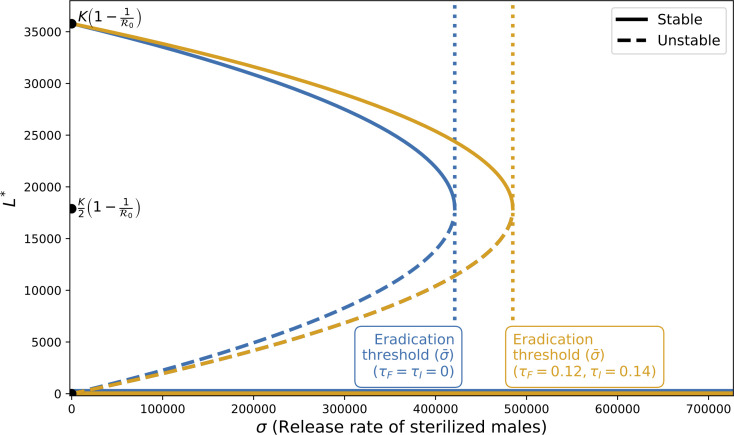
Bifurcation diagram showing the larval density at equilibrium L^*^ as a function of the sterilized male release rate σ. The equilibria of system (Eq 2) correspond to the intersection points between the function $\zeta (L^{*})$ (Eq 8) (blue, *First* scenario = Without MM, when $\tau_{F}=\tau_{I}=0$, or orange, *Last* scenario, when $\tau_{F}$ and $\tau_{I}>0$) and the σ constant. Stability is represented by solid lines, and unstable equilibria are shown by dashed lines. The *eradication thresholds* for each case are also shown (blue or orange dotted lines). The diagram was generated by setting the parameters to the values listed in Table 2.

Releasing a rate of sterilized males beyond this threshold drives the system into the stability region of the pest-free equilibrium (Fig 2), theoretically ensuring successful pest control. Mathematically, the tipping point $\bar{\sigma }$ corresponds to a saddle-node bifurcation: as σ increases past $\bar{\sigma }$, the stable infestation equilibrium and the unstable intermediate equilibrium collide and disappear, leaving only the pest-free equilibrium as stable. This explains why outcomes are effectively all-or-nothing: below $\bar{\sigma }$, control depends on the initial pest density, whereas above $\bar{\sigma }$, the pest population is guaranteed to collapse.

### Summary: equilibria and local stability

(i) If ${ℛ}_{0}\leq 1$ or $\sigma >\bar{\sigma }$ (Fig 2), the pest-free equilibrium is the unique equilibrium of the system (2) and is locally asymptotically stable.
(ii) If ${ℛ}_{0}>1$ and $\sigma <\bar{\sigma }$ (Fig 2), the system (Eq 2) admit three equilibria: the pest-free equilibrium which is locally asymptotically stable, the infestation equilibrium $E_{1}^{*}$ such that $L^{*}<\frac{K}{2}(1-\frac{1}{{ℛ}_{0}})$ which is unstable, and, the infestation equilibrium $E_{2}^{*}$ such that $L^{*}>\frac{K}{2}(1-\frac{1}{{ℛ}_{0}})$ which is locally asymptotically stable.

### 2.2 Agent-based model: Impact of sperm use bias on SIT effectiveness

While the compartmental model provides a population-level understanding of how multiple mating and sterilized male releases shape pest dynamics, it cannot fully capture individual-level variability or alternative sperm-use strategies. To explore these aspects in more detail, we turn to an agent-based modeling approach.

ABMs have previously been developed to study SIT against fruit flies. For example, Manoukis and Hoffman (2014) [89] implemented an individual-based demographic model to predict the time required for the eradication of *Ceratitis capitata*, helping to evaluate the adequacy of quarantine durations. Lux and Colacci (2025) [90] adapted an agent-based model to simulate SIT at a small scale, showing that local landscape structure strongly influences the behavior of wild and sterilized males, and consequently the effectiveness of releases. Finally, Diouf et al. (2022) [91] explored a boosted SIT approach, in which sterilized males carrying a pathogen transmit a biocide to *Bactrocera dorsalis* populations, significantly reducing fly densities and crop losses. Collectively, these examples illustrate how ABMs allow fine-grained studies of intervention impacts and the adaptation of control strategies to local biological and environmental conditions. Building on these developments, we adopt an agent-based approach to specifically explore the effects of sperm use biases on SIT efficiency, extending beyond the extreme cases considered in compartmental models.

#### 2.2.1 Agent-based model description.

As part of this study, an agent-based model was developed to simulate the reproductive dynamics of *D. suzukii* females under SIT deployment context. The model, implemented in Python using the Mesa package [99], operates in discrete time steps (one step corresponds to one day) and assumes a spatially well-mixed population. A brief description of the model is provided in the text, but the full ODD-based (Overview, Design concepts, and Details) description is provided in S5 Text to ensure clarity and reproducibility. In this model, we maintain similar assumptions to those established in the previous compartmental model, but we explore more scenarios of sperm use bias.

**Fig 3:**
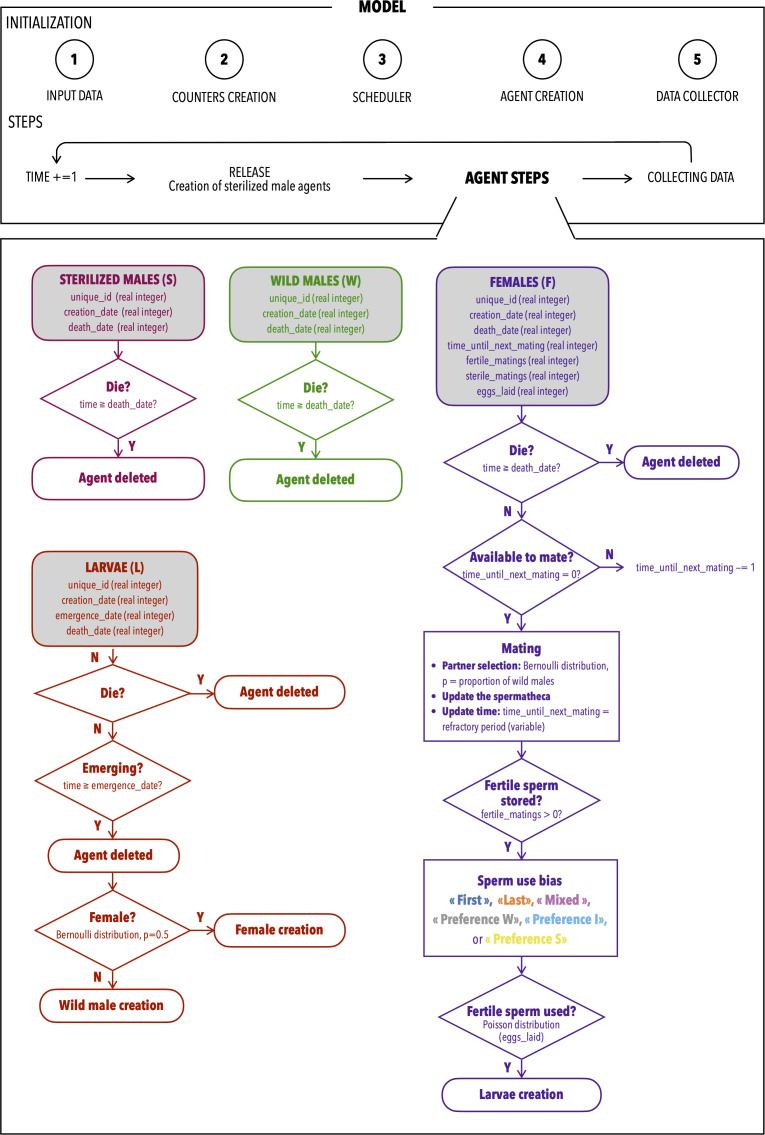
Flowchart describing the steps of the agent-based model. The top black rectangle outlines the general model framework, beginning with the initialization phase: (1) input of data (biological data linked to the life cycle, release quantity), (2) creation of counters to track population densities, (3) implementation of the scheduler, (4) creation of agents at the initial time step (four types): sterilized males, wild males, larvae, and females, and (5) parametrization of the data collector to specify the data to be recorded at each step. The subsequent steps, executed at each time step, include: advancing the simulation time, releasing sterilized males (creating new sterilized male agents), performing agent-specific steps for each category, collecting data, and repeating the cycle with the next time step. The bottom rectangle illustrates, in algorithmic diagrams, the specific steps for each type of agent: sterilized males (pink), wild males (green), larvae (red), and females (purple). These diagrams summarize the sequence of decisions and events for each agent category. The agent-based model is described in further details in Section 2.2.

The model includes four types of agents (Fig 3): wild males, sterilized males, females, and larvae (a compartment encompassing eggs, larvae, and pupae). Each agent is characterized by a set of biological traits such as longevity and mortality. Additionally, larvae have an emergence time, while females are also defined by their fecundity (number of eggs laid), their post-mating refractory time, and their ability to store and use sperm from multiple mates.

A central feature of the model is indeed the explicit representation of polyandrous female behavior. Females may mate multiple times during their lifetime, accumulating sperm from different males. The use of this sperm follows one of six predefined scenarios:

- ***First* = Without MM**: exclusive use of the sperm from the first mate, equivalent to a case without multiple mating;
- ***Last***: exclusive use of the sperm from the last mate;
- ***Mixed***: sperm use according to the proportion of fertile and sterilized sperm stored;
- ***Preference W*** (W for **Wild**): complete preference for fertile sperm, if available;
- ***Preference I*** (I for **Intermediate**): relative preference for fertile sperm over sterilized sperm. In this study: 80% preference (arbitrary value) for fertile sperm over sterilized sperm, when both are stored;
- ***Preference S***: complete preference for sterilized sperm, if available.

These scenarios directly influence the probability that a given egg is fertilized by a fertile versus a sterilized male, and thus affect population growth. In the absence of a definite answer of which sperm use scenario is the most relevant for *D. suzukii*, we opted to cover a wide range of options, ranging from reasonable ones to an highly improbable one (Preference S, which we put as a reference point as the most favourable situation for SIT).

The model also allows a flexible configuration of sterilized male release strategies, including the timing and intensity of releases, to investigate their combined effect with female re-mating behavior.

## 3 Results

### 3.1 Impact of multiple mating

#### 3.1.1 Definition of scenarios.

We define two distinct scenarios for multiple mating (MM):

**The *last* scenario**. In this scenario, females can re-mate and sperm from the last mating takes precedence over earlier sperm, so the female status (fertilized or infertile) and the production of offspring are determined by the last male the female mated with. This is the default scenario in the reduced model (Eq 2) with $\tau_{F}>0$ and $\tau_{I}>0$, where fertilized females (*F*_*F*_) continue to lay eggs until they mate with a sterilized male and transition into the infertile female compartment (orange in Fig 2).

**The *First* Scenario (= Without MM)**: In this scenario, the production of offspring is determined exclusively by the sperm of the first male the female mates with, regardless of any subsequent matings. This scenario also represents the absence of multiple mating and corresponds to model (2), where parameters $\tau_{I}=\tau_{F}=0$. It ensures that females, once fertilized, remain in that compartment (*F*_*F*_) and continue to lay eggs throughout their lifespan (blue in Fig 2).

#### 3.1.2 Asymptotic behavior.

**Fig 4:**
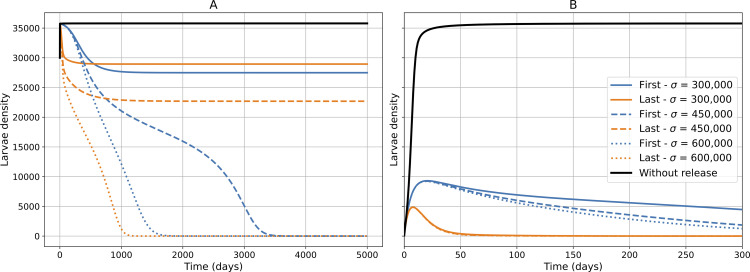
Simulations of larval density *L* over time, obtained by numerically simulating the system of equations in (Eq 2), for different initial infestation levels and sterilized male release rates (σ). **(A)** shows a high initial infestation level (initial conditions: *L* = 30,000, *M* = 100,000, *F*_*I*_ = 0, *F*_*F*_ = 100,000, $S=\frac{\sigma }{\mu_{S}}$, population almost at the no-SIT equilibrium), while **(B)** shows a low initial infestation level (initial conditions: *L* = 100, *M* = 300, *F*_*I*_ = 0, *F*_*F*_ = 300, $S=\frac{\sigma }{\mu_{S}}$) at the beginning of the SIT treatment. The black curves represent no sterilized male releases (σ=0). Blue curves show the *First* sperm use scenario ($\tau_{I}=\tau_{F}=0$), and orange curves show the *Last* scenario ($\tau_{I}>0$ and $\tau_{F}>0$). Line styles distinguish the different release rates: solid lines correspond to σ=300,000 (below the *eradication thresholds* in Fig 2), dashed lines to σ=450,000 (between the *eradication thresholds* in Fig 2), and dotted lines to σ=600,000 (above the *eradication thresholds* in Fig 2).

The asymptotic behavior is quite similar for both the *First* and *Last* scenarios (Fig 2). Indeed, three distinct cases can be identified based on the initial infestation level and the sterilized male release effort: (1) a high initial infestation with a small release rate, (2) a low initial infestation with a small release rate, and (3) a sufficiently high release rate. These three cases are illustrated in Fig 4. These patterns reflect the underlying saddle-node bifurcation at $\bar{\sigma }$, where below this threshold the system can exhibit either partial or minimal control depending on initial larval density, and above it, the pest-free equilibrium becomes the only stable state, corresponding to full eradication.

To make these cases explicit, we specify the initial conditions used in the simulations as follows: high initial infestation, with *L*(0)=30,000, *M*(0)=100,000, *F*_*I*_(0) = 0, and $F_{F}(0)=100,000$ (population close to the no-SIT equilibrium); and low initial infestation, with *L*(0)=100, *M*(0)=300, *F*_*I*_(0) = 0, and *F*_*F*_(0) = 300. This latter case reflects the early stage of an infestation in a strawberry tunnel, where only a few individuals have recently colonized the crop and the population remains far from its carrying capacity.

The simulations reveal that, for a high initial infestation level of pests at the start of SIT treatment, when the release rate σ is below the eradication thresholds for the *First* and *Last* scenarios (Fig 2, case (1)), the larval population is reduced compared to the “Without Release” scenario (*WR*) but remains at a relatively high level (Fig 4A). At intermediate release rates between *eradication thresholds*, population control is achieved without multiple mating, but maintained when multiple mating is present. For release rates σ exceeding the *eradication thresholds*, the larval populations are effectively controlled in both the *First* and *Last* scenarios (Fig 4A, case (3)). For a low initial infestation level of pests, the larval population is successfully controlled across all three ranges of release rates σ (Fig 4B), which corresponds to cases (2) and (3).

In the long term, the pest population seems easier to control in the *First* scenario (= Without MM) rather than in the *Last* scenario (Fig 4). This observation is supported by two arguments: (1) The locally asymptotically stable infestation equilibrium is slightly higher in the *Last* case (Fig 2). (2) The *eradication threshold* for the *Last* scenario is higher than for the *First* scenario (Fig 2). A higher sterilized male release rate σ will be required to control a population where females mate multiple times and use the last sperm (*Last*). When the release rate σ is between the *eradication thresholds* of the *First* and *Last* scenarios (σ=450,000, Fig 2), the larval population is eradicated under the *First* scenario, whereas it persists at a controlled level under the *Last* scenario (Fig 4A). For context, these eradication thresholds correspond to a sterilized-to-wild male ratio of approximately 65 for the *First* scenario and 74 for the *Last* scenario. These ratios are calculated by dividing the equilibrium number of sterilized males at the threshold by the equilibrium number of wild males.

#### 3.1.3 Transient dynamics.

During the transient regime, i.e., the short term, it can be observed that the population decreases much more quickly at the beginning of the simulation in the *Last* scenario (Fig 4A). Indeed, with fertilized females and larvae as initial conditions and by examining the model equations (Eq 2), in the *Last* scenario, we observe that fertilized females (*F*_*F*_) can transition directly into the infertile female compartment (*F*_*I*_) upon encountering sterilized males, due to the large number of sterilized males released from the initial time. In contrast, in the *First* case, fertilized females *F*_*F*_ cannot directly become “infertile”; there is a delay. These females will produce larvae *L*, and they will start doing so from the beginning of the simulation.

**Fig 5:**
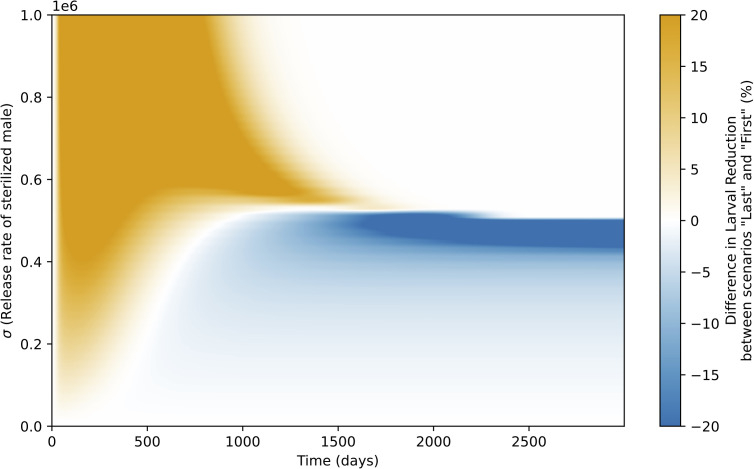
Heatmap showing the difference in larval density reduction between the *Last* and *First* scenarios, relative to a no-SIT situation (WR), across time (days, horizontal axis) and sterilized male release rates σ (vertical axis). Positive values (orange tones) indicate greater reduction in the *Last* scenario; negative values (blue tones) indicate better performance of the *First* scenario. Simulations were run over 3000 days under a high-infestation setting (initial conditions: *L* = 30,000, *M* = 100,000, *F*_*I*_ = 0, $F_{F}=100,000$). Parameters as in Table 2.

To further explore the impact of re-mating dynamics on control efficiency over time, we computed the difference in larval density reduction proportion between the *Last* and *First* scenarios, relative to a no-SIT baseline (*WR*) ($\frac{L_{First}-L_{Last}}{L_{WR}}$), as a function of time and sterilized male release rate σ (Fig 5). Simulations shown in this figure correspond to the high initial infestation case, with initial conditions *L*(0)=30,000, *M*(0)=100,000, *F*_*I*_(0) = 0, and $F_{F}(0)=100,000$, as defined previously. This visual comparison highlights the transient advantages of the *Last* scenario, especially during the initial deployment period. When release rates are moderate, the *Last* scenario achieves a sharper initial decline in larval density due to the immediate sterilization of fertilized females, whereas in the *First* scenario, a delay is introduced by the time required for offspring maturation and secondary mating events. However, this advantage tends to fade over time, and in both scenarios, sufficiently high release rates lead to effective long-term suppression, minimizing differences. Conversely, at low release rates, SIT impact is limited in both cases, and differences remain marginal. Interestingly, for a narrow range of intermediate release intensities, the *First* scenario becomes more effective in the very long term, likely due to its slower yet more stable impact on population dynamics. This heatmap thus helps clarify how mating dynamics interact with release intensity to influence SIT efficiency across different timescales.

Thus, in the short term (e.g., a timeframe of about 100 days, corresponding to the strawberry harvest period), multiple mating can be advantageous, as long as $\tau_{F}$ is not close to zero, meaning that not only females mated with sterilized males re-mate.

### 3.2 Impact of refractory rates

The *eradication threshold* (Eq 4) depends on the refractory rates $\tau_{F}$ and $\tau_{I}$. These rates correspond to the inverse of the time during which females are unable to mate again. When the refractory rates are independent of whether the female mated with a wild or a sterilized male ($\tau_{F}=\tau_{I}$), the *eradication threshold* is the same as without re-mating ($\tau_{F}=\tau_{I}=0$). However, when females re-mate faster after a sterilized male than after a wild male ($\tau_{I}>\tau_{F}$), as illustrated in Fig 2, the *eradication threshold* increases, and vice versa when they re-mate slower.

**Fig 6:**
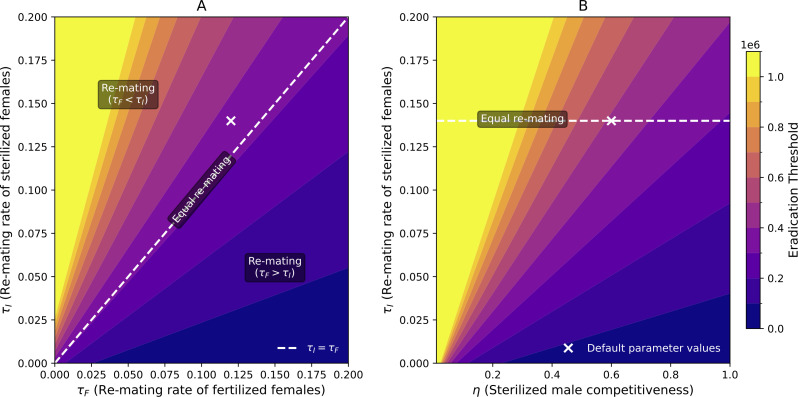
Heatmaps of the *eradication threshold* (Eq 4) as a function of (A) refractory rates $\tau_{F}$ (x-axis) and $\tau_{I}$ (y-axis) and (B) the competitiveness of sterilized males η (x-axis) and the refractory rate of females mated with sterilized males $\tau_{I}$ (y-axis). Default parameter values are represented by white crosses on heatmpas. **(A)** The parameter η is fixed at 0.6, as listed in Table 2, and the white dashed line represents the scenario of equal re-mating ($\tau_{F}=\tau_{I}$). The top-left region corresponds to $\tau_{F}<\tau_{I}$, while the bottom-right region corresponds to $\tau_{F}>\tau_{I}$. **(B)** The parameter $\tau_{F}$ is fixed at 0.12, as detailed in Table 2. The eradication threshold was capped at one million; values exceeding this limit are included in the yellow area of the heatmap.

The impact of re-mating in the *Last* scenario can therefore be examined according to the difference between refractory rates according to the nature of the last male the female mated with. So three cases emerge (see Fig 6A):

- When $\tau_{I}=\tau_{F}$, females re-mate at the same rate regardless of whether they were previously mated with a wild or a sterilized male. In this case, in the long term, re-mating has a neutral effect on the control strategy, as function $\zeta (L^{*})$ (Eq 3) is then independent of the refractory rates.
- When $\tau_{I}>\tau_{F}$, females previously mated with sterilized males re-mate faster than when mated with wild males. This scenario is the most detrimental to control efforts, as it increases the chances that females will be fertilized by wild males, thereby raising the *eradication threshold*.
- Conversely, when $\tau_{F}>\tau_{I}$, females previously mated with wild males re-mate faster than when mated with sterilized males. This scenario tends to facilitate population suppression since wild males have a higher probability of being replaced in subsequent matings. Biologically this case seems less realistic.

However, the two refractory rates ($\tau_{I}$ and $\tau_{F}$) do not exert the same influence on the *eradication threshold* (Fig 6A). When $\tau_{F}>\tau_{I}$, the *eradication threshold* is relatively insensitive to changes in $\tau_{F}$ and, to a lesser extent, in $\tau_{I}$. In contrast, when $\tau_{I}>\tau_{F}$, the *eradication threshold* becomes highly sensitive to even small increases in $\tau_{I}$ and $\tau_{F}$, especially when the re-mating rates are small, which can cause the required release ratio of sterilized males to reach impractical levels, making effective population control impossible to achieve. So re-mating rates tend to increase the *eradication threshold* but there are interactions between the two parameters.

The *eradication threshold* depends not only on the refractory rates but also on the competitiveness η of sterilized males (Fig 6B). Recall that when η=0, sterilized males are not competitive at all, whereas when η=1, sterilized males are as competitive as wild males. Importantly, the interaction between the re-mating rate and the competitiveness of sterilized males is not simply linear. While the *eradication threshold* is highly sensitive to variations in $\tau_{I}$, it is much less affected by changes in η, provided η remains sufficiently high. As expected, the most advantageous case for control, where the *eradication threshold* is the lowest, occurs when sterilized males are as competitive as wild males (η=1) and when females previously mated with a sterilized male re-mate as little as possible ($\tau_{I}$ close to 0, as they remain unavailable and “sterilized”) (Fig 6B). We observe that the interaction between sterilized male competitiveness η and the refractory rate $\tau_{I}$ influences the *eradication threshold*. Specifically, to return to biological values, when competitiveness exceeds the fixed value of 0.6, variations in $\tau_{I}$ have only a minor effect on the *eradication threshold* (Fig 6B). In contrast, for lower values of competitiveness, $\tau_{I}$ plays a more significant role. However, even with low re-mating rates, η must still be sufficiently high to prevent the *eradication threshold* from reaching impractical levels that would require unattainable release ratios of sterilized males.

### 3.3 Impact of sperm use

The previous section highlighted that female polyandry has a significant impact on the release efforts required for efficient SIT deployment. Furthermore, a question still remains unsolved: how are the population dynamics affected by multiple mating in cases of complex biases in the use of sperm beyond the extreme cases of using only the sperm of the *First* or *Last* mating? To answer this question within a pertinent timescale and in an agricultural context with non-perennial crops, we developed an agent-based model (ABM) to explore how potential sperm use biases impact pest control with SIT, focusing on proxies of SIT efficiency on a short timescale.

**Fig 7:**
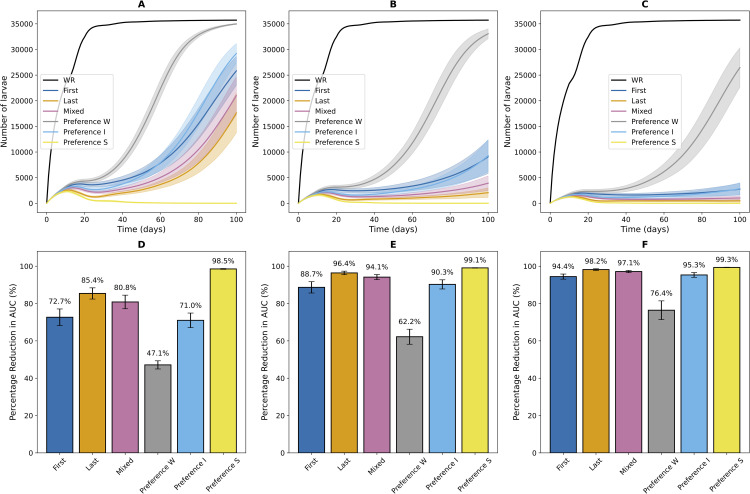
Dynamics of larval densities (A-B-C) and percentage reduction in areas under the curves (D-E-F) for different sperm bias and three sterilized male release rates σ: (A-D) σ=10,000, (B-E) σ=15,000, (C-F) σ=20,000. **(A–B–C)** Mean larval density over time across 100 simulations, with shaded areas representing standard deviation. The color code for sperm use scenarios is as follows: *WR* (black) represents the reference case Without Release (no sterilized males released), *First* (dark blue) corresponds to females using sperm from the first male they mated with, *Last* (orange) represents females using sperm from the last male they mated with, *Mixed* (pink) assumes a proportional use of fertile sperm linked to the proportion of eggs laid, and *Preference* reflects female preference for a specific sperm category, with three subcases: *Preference W* (gray) indicates total preference for fertile sperm, *Preference I* (light blue) indicates intermediate preference for fertile sperm, and *Preference S* (yellow) indicates total preference for sterilized sperm. **(D-E-F)** Histograms showing the percentage reduction in areas under the curves for the sperm use scenarios described in A-B-C, relative to the reference scenario *WR* without release, over a 100-day period. The error bars in these graphs represents the 95% confidence interval (CI 95%). Simulations were conducted under the same initial conditions: 1000 wild males, 1000 females, and 0 larvae. Results are based on 100 simulations performed with the agent-based model described in Section 2.2.

Simulation results highlight how different sperm use biases influence larval density and SIT success. The number of larvae was chosen as the indicator of SIT success because they are responsible for a large proportion of fruit damage. Fig 7 illustrates larval population dynamics and cumulated population reduction, compared to a situation without SIT (WR), under different sperm use biases (colors) and sterilized male release intensities (A–C panels).

Simulation results, obtained for low infestation levels, corresponding to an early phase in a strawberry tunnel, where the fruits are not yet infested and adults start being attracted to the resource and infesting the tunnel, show a strong short-term efficacy of SIT, with no pattern differences between sperm use biases at the beginning of the season (Fig 7). Increasing the number of sterilized males released consistently enhances SIT effectiveness, leading to high larval population reduction. Even in the least favorable scenario for SIT, where females prefer fertile sperm when stored (*Preference W*), a substantial increase in release effort allows for nearly complete larval suppression.

A noticeable difference appears between the *First* and *Last* scenarios (σ between 10,000 and 20,000 in Fig 7). The *Last* scenario, where females use sperm from their latest mate, proves more effective in reducing larval density than the *First* scenario. However, this difference diminishes as the number of released sterilized males increases, confirming trends observed in the compartmental model (Fig 5).

The *First* and *Preference I* scenarios yield very similar results, which is interesting given that the former represents a situation with no re-mating, while the latter allows re-mating but with a preference for fertile sperm (here set at 80%), a more biologically realistic setting. The *Mixed* scenario leads to intermediate larval reduction levels between the *First* and *Last* scenarios.

**Fig 8:**
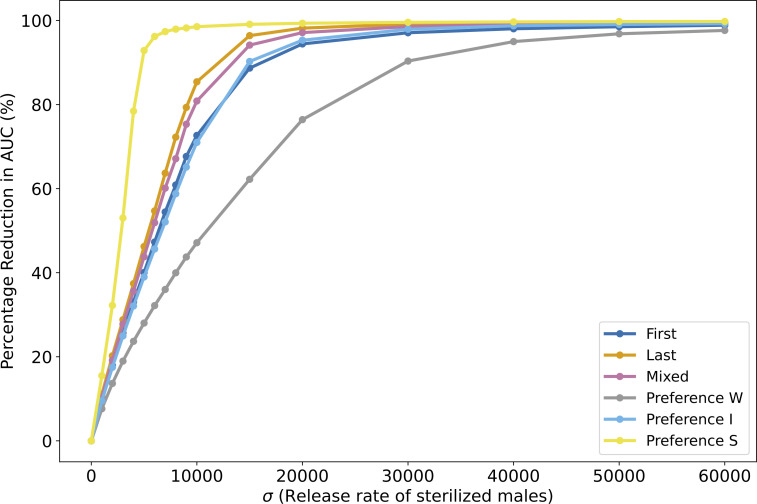
Graph showing the percentage reduction in areas under the curve (AUC) over a 100-day period, for different sperm use scenarios, relative to the reference scenario *WR* without release (no sterile insect technique applied) as a function of sterile male released rates. Simulations were conducted under the same initial conditions: 1000 wild males, 1000 females, and 0 larvae. Each point is the average over 100 simulations performed with the agent-based model described in Section 2.2.

Overall, at sufficiently high release rates, differences between sperm use strategies become less pronounced, with SIT achieving successful larval population reduction across all tested scenarios (see Fig 8).

In addition, simulation results, obtained for high infestation levels, with initial conditions including fertilized females and larvae, reproducing an early-season infestation scenario in a tunnel where mated flies and larvae are already present in some fruits, reveal a dynamic inversion over time between sperm use scenarios, consistent with findings from the compartmental model (see Sect. 3.1.3). In particular, the *Last* scenario shows a faster initial decline in larval density compared to *First* (= Without MM), but may lead to less effective suppression in the long term if release rates are not sufficiently high. This temporal shift in effectiveness is visible as in the compartmental model (details in S6 Text), reinforcing the robustness of this pattern across modeling approaches. However, this result remains dependent on the initial conditions and thus on the simulated starting context: for example, in the hypothetical and rather unrealistic case where the simulation would start only with larvae and no adults, the dynamics with and without re-mating would be equivalent, with no delay effect.

## 4 Discussion

The sterile insect technique (SIT) is a species-specific method capable of suppressing pest populations thanks to release of sterilized males. Our results first confirm this strong potential. In the compartmental model, this is evidenced by the bifurcation structure (Fig 2), where sufficiently high release efforts drive the system toward the pest-free equilibrium, which becomes the only stable state. Consistently, simulations (Fig 4) show that large releases rapidly reduce population densities across a wide range of initial conditions, highlighting the robustness of SIT as a control strategy. However, this global potential does not automatically translate into success in all biological contexts. In particular, mating dynamics may substantially disrupt the efficiency of control efforts.

Our results highlight how multiple mating and sperm use biases influence SIT outcomes. In our study, the compartmental model offers a rapid and intuitive understanding of global dynamics, whereas the agent-based model captures more nuanced biological mechanisms and stochastic variability. Used together, they provide a more comprehensive framework for evaluating the impact of sperm use biases on SIT outcomes. Regarding *Drosophila suzukii*, in the long term, polyandry is disadvantageous for SIT effectiveness compared to a case without polyandry, as it requires additional sterilized male releases to control pest populations (Figs 2 and 5). It thus makes SIT more costly to implement. Indeed, the existence of re-mating behavior raises the *eradication threshold*, as females previously mated with sterilized males re-mate more, requiring more sterilized males for effective population control. Regarding the realism of the numerical values obtained for the *eradication threshold*, for instance, without re-mating (“First” scenario, Fig 2), we obtain an approximate value of 420,000. This corresponds to an equilibrium population of sterilized males of 420,000 / 0.054. Dividing this by the equilibrium number of wild males, which is around 120,000, gives a sterilized-to-wild male ratio of about 65. With multiple mating (“Last” scenario, Fig 2), we obtain an approximate value of 480,000, giving a sterilized-to-wild male ratio of about 74. Ayala et al. (2025) [100] report that a 40:1 sterile-to-fertile male ratio is sufficient to reduce by 80% pupae production. Therefore, these numerical values do not appear aberrant for achieving eradication, emphasizing that re-mating behavior strongly affects the release effort needed. In contrast, polyandry can be beneficial in the short term, such as during a single non-perennial fruit season, by further reducing pest populations. However, the difference between sperm use biases remains limited. These results depend on the parameters, particularly the refractory rates and sterilized male competitiveness, which influence the release effort required.

Intuitively, if only females that previously mated with a sterilized male re-mate, we can expect that re-mating would complicate the effectiveness of SIT. However, if refractory rates are similar regardless of the female previous mate (wild or sterilized male), multiple mating can be beneficial in the short term (Fig 5). Indeed, when fertilized females are already present, the transition between reproductive statuses occurs more rapidly when females re-mate, whereas without re-mating, a full generation is needed before a noticeable shift in the proportion of offspring sired by sterilized males occurs. These results highlight the importance of reproductive dynamics in SIT efficiency: effectiveness is not determined solely by the quantity of released males but also by their ability to engage in mating. Our findings align with previous studies, such as Barclay [101], which has explored the implications of multiple mating on SIT. Barclay [101] presented a model assuming strict female monogamy and compared it to a scenario where females frequently mate. He found that the system is easier to control under monogamy, particularly when mating propensity is low, although eradication takes longer. Furthermore, stability does not seem to be affected by mating frequency in his model. Additionally, Dyck et al. [1] discussed how multiple mating complicates control but does not disqualify a pest species as a candidate for SIT. While this review covers various studies on SIT, it does not highlight cases where multiple mating is explicitly beneficial. Instead, it suggests that while multiple mating may introduce complications, it does not render SIT ineffective. Our study provides a more unexpected perspective: in the short term, multiple mating can actively contribute to the success of SIT, rather than merely being a neutral or complicating factor. We demonstrate that the species’ reproductive mode differentially affects SIT efficiency depending on the release strategy and the timescale considered (Fig 5). Over the long term, polyandry reduces SIT efficiency, whereas over the short term, female re-mating can enhance it. Most modeling studies on this topic focus on asymptotic dynamics and fail to identify polyandry as an opportunity [9,101]. It would be of considerable interest to assess whether the short-term advantage we highlight persists in alternative frameworks, particularly in models incorporating more complex mating and re-mating sequences [9,83].

Our simulations of the agent-based model reveal that the impact of different sperm use biases varies depending on the release effort of sterilized males. Quantitatively, over a range of release rates (from 10,000–60,000 sterilized males released) within an arbitrarily defined season, the larval population is reduced by 43.7% to 97.6% when females preferentially use fertile sperm, the most unfavorable scenario, whereas it is reduced by 79.3% to 99.7% when females utilize the last sperm, a more favorable scenario. These percentages refer to the cumulative number of larvae produced over a 100-day period, relative to the case without SIT. This highlights a strong short-term effectiveness of pest reduction through increased release efforts, regardless of the sperm use bias tested (see Fig 8). However, scenarios where females favor fertile sperm or without multiple mating tend to reduce the short-term effectiveness of SIT. Contrary to our initial hypothesis, which assumed that the sperm use bias would have a strong impact on the ability of SIT to suppress populations, and to what is suggested in the literature [67], the quantitative difference in SIT effectiveness depending on sperm use bias remains limited.

The biases in sperm use were arbitrarily defined in our simulations but may correspond to natural variability in sperm storage and use. *D. suzukii* females possess two spermathecae [69] and can produce offspring from up to five different males [68]. Some data suggest a slight preference for the sperm from the first mating [92], while others indicate that mating duration is positively correlated with male ejaculate investment [76], influencing sperm use probability in polyandrous contexts [77]. If sterilized males transfer less sperm due to shorter mating, females are more likely to preferentially use fertile sperm simply because it is more abundant. The first mating duration has been observed to be shorter than the second one [92], and irradiation is known to alter the quantity of sperm transferred to females [67]. Additionally, we cannot exclude the possibility of a more active mechanism, where females might “recognize” and react to the mating status of their partner, as suggested by research on *Wolbachia*-infected males.

In fact, multiple mating and sperm use biases are not only relevant to the success of SIT but also impact other pest control strategies, such as those involving *Wolbachia*. These factors directly influence reproduction dynamics, bacterial spread, and the efficiency of population replacement or suppression methods. For instance, *Wolbachia* infection alters female receptivity to mating and re-mating frequency, particularly after mating with an infected male [102,103]. He et al. (2018) showed that females mated with infected males exhibited lower refractory rate, potentially enhancing *Wolbachia*-based population replacement strategies [102]. Conversely, Osorio et al. (2023) found that females, after mating with infected males, showed an increased tendency to re-mate, potentially undermining population replacement efforts if re-mating occurs with uninfected males [103]. These findings emphasize the need for a deeper understanding of mating dynamics to optimize pest population control strategies effectively [104–110].

To conclude, our findings highlight the critical role of female re-mating dynamics in shaping the effectiveness of SIT strategies. In particular, rapid re-mating following copulation with a sterilized male, combined with biased sperm use in favor of fertile males, can drastically increase the number of sterilized males required, sometimes beyond operational feasibility. While improving sterilized male competitiveness remains essential, better understanding and integrating the biological determinants of re-mating (e.g., refractory period variability, sperm selection mechanisms) could open new avenues to optimize SIT deployment. In fact, additional experiments on female re-mating with sterilized males would be valuable to further inform and validate model assumptions. In this context, moving beyond eradication to short-term population suppression strategies, particularly in seasonal crops, offers a pragmatic path forward. Future models should also account for damage caused by sterile oviposition, which remains a critical factor when evaluating the environmental impact of female mating behavior on SIT efficiency.

## Supporting information

S1 Text Description of biological parameters and data sources for the compartmental and agent-based models. (PDF)

S2 Text Model reduction using Tikhonov’s theorem, derivation of the reduced system. (PDF)

S3 Text Analysis of the pest-free equilibrium. (PDF)

S4 Text Analysis of infestation equilibria. (PDF)

S5 Text Agent-based model description with ODD protocol. (PDF)

S6 Text Comparison between the compartmental model and the agent-based model. (PDF)
