# Supplementary material for: Polyandry: A threat or an opportunity for the sterile insect technique?
Source: PLoS Comput Biol. 2026 Apr 29;22(4):e1014212. doi: 10.1371/journal.pcbi.1014212 (PMC13143183; doi:10.1371/journal.pcbi.1014212)
Supplement: S1 Text — (PDF) [file pcbi.1014212.s001.pdf]

S1 Description of Biological Parameters and Data Sources

S1.1 Details on the compartmental model parameters

The biological parameters used to model *Drosophila suzukii* were estimated from several studies and datasets and reported in Table 2.

Estimation of the larval mortality rate We assume that the survival probability of immature sub-stages (eggs, larvae and pupae) can be approximated by an exponential decay function:

$$\phi(t) = e^{-\mu_L t},$$

where μ_L is a constant mortality rate, and t is the development time (in days). We aim to estimate the overall survival probability from egg to adult emergence.

To do so, we used stage-specific survival probabilities extracted from the study by Emiljanowicz et al. [96], where the authors provide the proportion of individuals alive at age x that survive through each developmental interval. Specifically, the proportion of individuals surviving the egg stage is 0.868. The larval stage is divided into three substages, with survival proportions of 0.913, 0.952, and 0.900 respectively. Finally, the survival proportion during the pupal stage is 0.944.

To compute the total survival probability from egg to adult, we multiply the proportions across all sub-stages:

$$\phi = 0.868 \times 0.913 \times 0.952 \times 0.900 \times 0.944 = 0.640.$$

The total development time from egg to adult is approximately 12 days, so mortality rate μ_L can be deduced as follows:

$$\mu_L = -\frac{\ln(\phi)}{t} = -\frac{\ln(0.640)}{12} = 0.037.$$

This estimation provides a biologically grounded value for μ_L used in the population model (Eq. 2).

Estimation of the adult mortality rate Female (μ_F) and wild male (μ_M) mortality rates were estimated from figure 4 of [97], focusing on the summer morph in the mainland condition. Using the R package `digitize` [104], we extracted the minimum, maximum, median, first quartile (Q1), and third quartile (Q3) values from the box plots for both females and males.

For females, the extracted longevity values were: minimum = 22.09, Q1 = 68.51, median = 92.82, Q3 = 101.66, and maximum = 143.86. For males, the corresponding values were: minimum = 29.83, Q1 = 60.77, median = 76.24, Q3 = 89.50, and maximum = 129.28.

To estimate the mean longevity, we applied the formula proposed by Wan et al. [105], which is appropriate when the sample size is between 15 and 70 (here sample size is equal to 50 for females and for males) and when the minimum, maximum, median, Q1, and Q3 are available. The formula used is:

$$\text{Mean} = \frac{\text{min} + 2 \times Q_1 + 2 \times \text{median} + 2 \times Q_3 + \text{max}}{8}$$

This yields estimated mean longevities of 86.5 days for females and 76.5 days for males. Mortality rates were then calculated as the inverse of the estimated longevities, leading to $\mu_F = 0.012$ and $\mu_M = 0.013$.

Remark: Interestingly, comparable adult mortality rates were estimated under laboratory conditions by Emilianowicz et al. (2014) [96], with $\mu_F = 0.012$ and $\mu_M = 0.011$. These results also show that female and male mortality rates are close.

Estimation of egg-laying rate, larval hatching rate and sex ratio The daily egg-laying rate (ω) was taken from Emiljanowicz et al. [96], which reported a mean production of 5.7 ± 0.24 eggs per day, rounded up to 6 in the parameter set. From egg to adult, individuals develop in 13 days, leading to a larval hatching rate ν of $\frac{1}{13} = 0.08$ days^{-1}. The sex ratio (p) was considered equal to 0.5, as indicated by [96].

Estimation of the sterilized male competitiveness The competitiveness of sterilized males (η) was calculated based on data from [98], which indicated that irradiated males achieved a mating success rate of 37.5%, compared to 62.5% for wild males. Parameter η in model (Eq. 2) represents the relative mating success of irradiated males compared to wild males, so $\eta = \frac{37.5}{62.5} = 0.60$.

Estimation of the carrying capacity (K) To estimate the carrying capacity K, we used data on strawberry production and larval infestation (source: Occitanie Chamber of Agriculture, https://occitanie.chambre-agriculture.fr). Over the course of a year, corresponding to approximately 100 days of production, the daily yield is about 0.036 kg/m^2, or 18 kg of strawberries per day in a 500 m^2 tunnel. Given that a strawberry weighs approximately 20 grams, this corresponds to around 900 strawberries produced daily in the tunnel.

According to experiments conducted by the CTIFL (Centre Technique Interprofessionnel des Fruits et des Légumes, personal communication), each strawberry contains on average 20 larvae, based on fruit sampling and larval counts [min = 0, max = 265]. Assuming that only ripe fruits can host larvae (i.e., fruits that are likely to be harvested), this results in approximately 18,000 larvae per day across the 500 m^2 production area.

Several studies have shown that *D. suzukii* females strongly prefer laying eggs in ripe fruits, which are typically the fruits harvested on the same day, and thus at the end of their maturation process. This oviposition preference is well documented by Karageorgi et al. [106], who demonstrated that *D. suzukii* evolved to target healthy, intact, ripening fruits, unlike most other Drosophila species that prefer decaying fruits. Although females can occasionally lay eggs in unripe or overripe fruits when no better options are available, such behavior remains marginal. Kienzle et al. [107] confirmed that even under conditions of limited resource availability, females still predominantly choose ripe, non-fermented fruits. These findings highlight a marked, though flexible, preference for ripe fruits as oviposition sites.

In our model, we assume that the resource (i.e., the number of ripe fruits available for oviposition) is constant over time. Since harvest takes place every two days, we consider that, at any given time, the resource available to the flies corresponds to twice the daily production.

Thus, the carrying capacity K, defined as the maximum number of larvae that can be supported by the available resource, is estimated to be:

$$K = 2 \times \text{daily number of strawberries} \times \text{average number of larvae per fruit}$$

$$K = 2 \times 900 \times 20 = 36{,}000 \text{larvae per tunnel per day.}$$

Estimation of the refractory rates Finally, the refractory rates for “fertilized” and “sterilized” females (τ_F and τ_I) were estimated from Table 1 of [93], which reports the

average refractory periods depending on the type of male with which the females previously mated. Specifically, the average refractory period was 8.25 days for females that mated with wild males and 7.40 days for females that mated with sterilized males. The refractory rates were obtained by taking the inverse of these average values, resulting in $\tau_F \approx 0.12$ days^{-1} and $\tau_I \approx 0.14$ days^{-1}.

S1.2 Details on the agent-based model parameters

All data extracted from the literature to calibrate the agent-based model are listed in Table A in S1 Text and detailed in the previous section. In addition, the table specifies the type of distribution used (uniform $\mathcal{U}$, Bernoulli $\mathcal{B}$) or if a fixed value was applied for each parameter.

Table A. Range of values used to estimate parameters for the agent-based model 2.2 on *Drosophila suzukii*

Parameter/Description	Value or Range	Unit	Reference
Sterilized male release rate	[0 - 60,000]	ind.days^{-1}.ha^{-1}	-
Sterilized male lifespan	$\mathcal{U}(1, 36)$	day	[25]
Sterilization cost	0.40	-	[98]
Wild male lifespan	$\mathcal{U}(31, 129)$	day	[97]
Average number of eggs laid	6	eggs per female per day	[96]
Mortality rate of eggs	0.13	day^{-1}	[96]
Mortality rate of larvae stage L1 (1-2 days)	0.087	day^{-1}	[96]
Mortality rate of larvae stage L2 (2-3 days)	0.048	day^{-1}	[96]
Mortality rate of larvae stage L3 (3-6 days)	0.10	day^{-1}	[96]
Mortality rate of pupae	0.056	day^{-1}	[96]
Larval emergence	$\mathcal{U}(10, 20)$	day	[96] [1]
Sex ratio	$\mathcal{B}(1, 0.5)$	-	[96]
Carrying capacity	36,000	larvae	Calculated [2]
Female lifespan	$\mathcal{U}(21, 144)$	day	[97]
Refractory period for fertilized females	$\mathcal{U}(3, 13)$	day	[93]
Refractory period for infertile females	$\mathcal{U}(4, 11)$	day	[93]

[1] In the literature, larval emergence occurs at 12.8 ± 0.2 days [96]. However, we chose a broader range to account for environmental variability.

[2] Carrying capacity is set to 36,000 larvae for a strawberry tunnel of 500 m^2. Larval growth follows a logistic model to account for density-dependent competition. The mean number of eggs laid per female agent per day is adjusted, to represent the fact that not all larvae will be able to develop, as follows: Eggs per female $=$ Average number of eggs laid $\times \left(1 - \frac{\text{larvae population size}}{\text{Carrying capacity}}\right)$.
