# Supplementary material for: Polyandry: A threat or an opportunity for the sterile insect technique?
Source: PLoS Comput Biol. 2026 Apr 29;22(4):e1014212. doi: 10.1371/journal.pcbi.1014212 (PMC13143183; doi:10.1371/journal.pcbi.1014212)
Supplement: S2 Text — (PDF) [file pcbi.1014212.s002.pdf]

S2 Model reduction

We adopt a slow-fast approximation approach used in ecology [108] to reduce the system dimension. We perform this reduction using Tikhonov's theorem [109], which applies to singularly perturbed systems where fast variables rapidly relax to a quasi-equilibrium while slow variables evolve. To justify this approach, we assume that mating occurs very rapidly when females are sexually mature and available [94], implying that χ is much larger than the other parameters. We define X such as $\chi = \frac{X}{\epsilon}$, with $0 < \epsilon \ll 1$. Model (Eq. 1) becomes:

$$\begin{cases} \dot{L} = \omega(1 - \frac{L}{K})F_F - (\mu_L + \nu)L, \\ \dot{M} = \nu p L - \mu_M M, \\ \dot{F_U} = \nu(1-p)L + \tau_F F_F + \tau_I F_I - \mu_F F_U - \frac{X}{\epsilon} F_U, \\ \dot{F_I} = \frac{X}{\epsilon}\frac{\eta S}{M+\eta S} F_U - (\tau_I + \mu_F)F_I, \\ \dot{F_F} = \frac{X}{\epsilon}\frac{M}{M+\eta S} F_U - (\tau_F + \mu_F)F_F, \\ \dot{S} = -\mu_S S + \sigma. \end{cases}$$

We also introduce a new variable:

$$G_U = \chi F_U = \frac{X}{\epsilon} F_U.$$

This change has the advantage that all fast terms involving χF_U now appear as G_U, simplifying the separation of time scales. The original model becomes:

$$\begin{cases} \dot{L} = \omega\left(1 - \frac{L}{K}\right) F_F - (\mu_L + \nu)L, \\ \dot{M} = \nu p L - \mu_M M, \\ \dot{G_U} = \frac{X}{\epsilon}\left[\nu(1-p)L + \tau_F F_F + \tau_I F_I - \mu_F \frac{\epsilon}{X} G_U\right] - \frac{X}{\epsilon} G_U, \\ \dot{F_I} = \dfrac{\eta S}{M + \eta S} G_U - (\tau_I + \mu_F)F_I, \\ \dot{F_F} = \dfrac{M}{M + \eta S} G_U - (\tau_F + \mu_F)F_F, \\ \dot{S} = -\mu_S S + \sigma. \end{cases}$$

Introducing the fast time-scale $\tilde{t} = \frac{t}{\epsilon}$ and defining L', M', F'_U, F'_I, F'_F, S' as the derivatives with respect to $\tilde{t}$, we obtain:

$$\begin{cases} L' = \epsilon\left[\omega\left(1 - \frac{L}{K}\right) F_F - (\mu_L + \nu)L\right], \\ M' = \epsilon(\nu p L - \mu_M M), \\ G'_U = X\left[\nu(1-p)L + \tau_F F_F + \tau_I F_I - G_U - \mu_F \frac{\epsilon}{X} G_U\right], \\ F'_I = \epsilon\left(\dfrac{\eta S}{M + \eta S} G_U - (\tau_I + \mu_F)F_I\right), \\ F'_F = \epsilon\left(\dfrac{M}{M + \eta S} G_U - (\tau_F + \mu_F)F_F\right), \\ S' = \epsilon(-\mu_S S + \sigma). \end{cases}$$

According to Tikhonov's theorem, if the fast subsystem has a unique and asymptotically stable equilibrium when slow variables are constant, the slow dynamics can be approximated by replacing the fast variable with this equilibrium. The variable G_U is fast, while L, M, F_I, F_F, S evolve slowly.

In the fast time-scale, letting ϵ tend to 0, the slow variables are approximately

constant, while G_U follows a linear ODE:

$$\begin{cases} L' = 0, \\ M' = 0, \\ G_U' = X\left[\nu(1-p)L + \tau_F F_F + \tau_I F_I - G_U\right], \\ F_I' = 0, \\ F_F' = 0, \\ S' = 0. \end{cases}$$

Thus, the fast subsystem has a unique and asymptotically stable equilibrium:

$$G_U = \nu(1-p)L + \tau_F F_F + \tau_I F_I.$$

Since $F_U = \frac{\epsilon}{X} G_U$, this also implies that F_U rapidly tends to zero.

By replacing the G_U equilibrium back into the slow system (for $\dot{F}_I$ and $\dot{F}_F$), we obtain the reduced model:

$$\begin{cases} \dot{L} = \omega\left(1 - \frac{L}{K}\right) F_F - (\mu_L + \nu)L, \\ \dot{M} = \nu p L - \mu_M M, \\ \dot{F}_I = \nu(1-p)\frac{\eta S}{M+\eta S} L + \tau_F \frac{\eta S}{M+\eta S} F_F - (\mu_F + \tau_I \frac{M}{M+\eta S}) F_I, \\ \dot{F}_F = \nu(1-p)\frac{M}{M+\eta S} L + \tau_I \frac{M}{M+\eta S} F_I - (\mu_F + \tau_F \frac{\eta S}{M+\eta S}) F_F, \\ \dot{S} = -\mu_S S + \sigma. \end{cases}$$

This reduced model captures the slow dynamics under the assumption that mating occurs almost instantaneously after females become available, with F_U rapidly relaxing to near zero.
