# Supplementary material for: Polyandry: A threat or an opportunity for the sterile insect technique?
Source: PLoS Comput Biol. 2026 Apr 29;22(4):e1014212. doi: 10.1371/journal.pcbi.1014212 (PMC13143183; doi:10.1371/journal.pcbi.1014212)
Supplement: S3 Text — (PDF) [file pcbi.1014212.s003.pdf]

S3 Analysis of the pest-free equilibrium E_0^*

S3.1 Equilibrium E_0^*

The pest-free equilibrium of system (Eq. 2) is given by:

$$E_0^* = (L^*, M^*, F_I^*, F_F^*, S^*) = (0, 0, 0, 0, \frac{\sigma}{\mu_S}).$$

S3.2 Stability analysis of E_0^*

We analyze the local stability of the pest-free equilibrium E_0^* by evaluating the Jacobian matrix of the (L, M, F_I, F_F) subsystem of the reduced model (Eq. 2) at $S = S^*$. The general Jacobian matrix is given by:

$$J = \begin{pmatrix} -\frac{\omega}{K}F_F - (\mu_L + \nu) & 0 & 0 & \omega(1 - \frac{L}{K}) \\ \nu p & -\mu_M & 0 & 0 \\ \nu(1-p)\frac{\eta S}{M+\eta S} & -\frac{\eta S}{(M+\eta S)^2}(\nu(1-p)L + \tau_F F_F + \tau_I F_I) & -(\mu_F + \tau_I \frac{M}{M+\eta S}) & \tau_F \frac{\eta S}{M+\eta S} \\ \nu(1-p)\frac{M}{M+\eta S} & \frac{\eta S}{(M+\eta S)^2}(\nu(1-p)L + \tau_F F_F + \tau_I F_I) & \tau_I \frac{M}{M+\eta S} & -(\mu_F + \tau_F \frac{\eta S}{M+\eta S}) \end{pmatrix} \quad (5)$$

At the equilibrium E_0^*, where $L = M = F_I = F_F = 0$, the Jacobian matrix becomes:

$$J(E_0^*) = \begin{pmatrix} -(\mu_L + \nu) & 0 & 0 & \omega \\ \nu p & -\mu_M & 0 & 0 \\ \nu(1-p) & 0 & -\mu_F & \tau_F \\ 0 & 0 & 0 & -(\mu_F + \tau_F) \end{pmatrix}$$

This matrix has a block triangular structure, which allows us to identify its eigenvalues directly from the eigenvalues of each block on the diagonal:

- The lower-right block $-(\mu_F + \tau_F)$ (isolated by the black vertical line) is an eigenvalue.
- The upper-left 3×3 block is lower-triangular and yields eigenvalues $-(\mu_L + \nu)$, $-\mu_M$ and $-\mu_F$.

Conclusion: Since all eigenvalues of $J(E_0^*)$ have strictly negative real parts, the pest-free equilibrium E_0^* is locally asymptotically stable. This conclusion holds regardless of the value of S^*.
