# Supplementary material for: Polyandry: A threat or an opportunity for the sterile insect technique?
Source: PLoS Comput Biol. 2026 Apr 29;22(4):e1014212. doi: 10.1371/journal.pcbi.1014212 (PMC13143183; doi:10.1371/journal.pcbi.1014212)
Supplement: S4 Text — (PDF) [file pcbi.1014212.s004.pdf]

S4 Analysis of infestation equilibria

S4.1 Equilibria of the subsystem

For the (L, M, F_I, F_F) subsystem at $S = S^*$, the equilibria of system (Eq. 2) are the solutions of the following equations. We introduce the notation $\gamma = \frac{\nu p}{\mu_M}$ to simplify expressions:

$$\Leftrightarrow \begin{cases} F_F^* = \frac{(\mu_L+\nu)}{\omega(1-\frac{L^*}{K})} L^*, \\ M^* = \frac{\nu p}{\mu_M} L^* = \gamma L^*, \\ F_I^* = \left(\frac{\nu(1-p)}{\mu_F} - \frac{(\mu_L+\nu)}{\omega(1-\frac{L^*}{K})}\right) L^*, \\ \nu(1-p)\frac{\eta S^*}{M^*+\eta S^*} L^* + \tau_F \frac{\eta S^*}{M^*+\eta S^*} F_F^* - (\mu_F + \tau_I \frac{M^*}{M^*+\eta S^*}) F_I^* = 0. \end{cases} \tag{6}$$

with F_I^* obtained from $\dot{F}_I + \dot{F}_F = 0$. Substituting F_I^* and F_F^* into the last equation, we obtain:

$$\nu(1-p)\frac{\eta S^*}{M^* + \eta S^*} L^* + \tau_F \frac{\eta S^*}{M^* + \eta S^*} \frac{(\mu_L + \nu)}{\omega(1 - \frac{L^*}{K})} L^* - \left(\frac{\mu_F(M^* + \eta S^*) + \tau_I M}{M^* + \eta S^*}\right)\left(\frac{\nu(1-p)}{\mu_F} - \frac{(\mu_L + \nu)}{\omega(1 - \frac{L^*}{K})}\right) L^* = 0.$$

Replacing M^* by its expression in (Eq. 6) and simplifying, we arrive at an equation that solely depends on the density of larvae L:

$$-\frac{\nu(1-p)\omega(\mu_F + \tau_I)\gamma}{K\eta\mu_F(\mu_L + \nu)(\mu_F + \tau_F)}(L^*)^2 + \frac{(\mu_F + \tau_I)(\nu(1-p)\omega - \mu_F(\mu_L + \nu))\gamma}{\eta\mu_F(\mu_L + \nu)(\tau_F + \mu_F)} L^* = S^*.$$

We define the basic reproduction number $\mathcal{R}_0 = \frac{\omega(1-p)\nu}{\mu_F(\mu_L+\nu)}$, and substitute it, along with $S^* = \frac{\sigma}{\mu_S}$, into the equation above to obtain:

$$-\frac{\gamma\mu_S(\mu_F + \tau_I)\mathcal{R}_0}{K\eta(\mu_F + \tau_F)}(L^*)^2 + \frac{\gamma\mu_S(\mu_F + \tau_I)(\mathcal{R}_0 - 1)}{\eta(\mu_F + \tau_F)} L^* = \sigma. \tag{7}$$

Thus, L^* is given by the intersection between the straight line $y = \sigma$ and the parabola $y = \zeta(L^*)$ 2, where:

$$\zeta(L^*) = \frac{\gamma\mu_S(\mu_F + \tau_I)}{\eta(\mu_F + \tau_F)}\left(-\frac{\mathcal{R}_0}{K}(L^*)^2 + (\mathcal{R}_0 - 1)L^*\right). \tag{8}$$

When $\sigma = 0$, the roots of (Eq. 8) are easy to deduce: $L^* = 0$ and $L^* = K(1 - \frac{1}{\mathcal{R}_0})$.

S4.2 Stability analysis of infestation equilibria

Case $\mathcal{R}_0 \leq 1$ The parabola $\zeta(L^*)$ admits a zero root and a non positive root. In this case, since the highest-degree term is negative, the equation $\zeta(L^*) = \sigma$ has no solution, and there is no infestation equilibrium.

Case $\mathcal{R}_0 > 1$ The parabola $\zeta(L^*)$ admits a zero root and a positive root. In this case, the equation $\zeta(L^*) = \sigma$ may have 2 or 0 solutions: E_1^* and E_2^*, which correspond to the points of intersection between the straight line $y = \sigma$ and the parabola $\zeta(L^*)$.

The summit of the parabola is reached for L^* in the middle of the two roots $\zeta(L)$, so that, when E_1^* and E_2^* exist, $L^*(E_1^*) < \frac{K}{2}(1 - \frac{1}{\mathcal{R}_0}) < L^*(E_2^*)$. Also numerical exploration of the Jacobian matrix (Eq. 5) of the (L, M, F_F, F_I) subsystem at $S = S^*$ of model (Eq. 2) shows that E_1^* is always unstable and E_2^* asymptotically stable. This analysis was performed for a range of release rates σ to explore the bifurcation structure of the system.
