# Supplementary material for: Polyandry: A threat or an opportunity for the sterile insect technique?
Source: PLoS Comput Biol. 2026 Apr 29;22(4):e1014212. doi: 10.1371/journal.pcbi.1014212 (PMC13143183; doi:10.1371/journal.pcbi.1014212)
Supplement: S5 Text — (PDF) [file pcbi.1014212.s005.pdf]

S5 Agent-based model description with ODD protocol

The model is described using aspects of the Overview, Design concepts, Details (ODD) protocol [110] and was implemented using Python 3.9.12 with the Mesa package [99], specifically designed for agent-based models. Numerical simulations were calibrated on the species *D. suzukii*.

S5.1 Overview

S5.1.1 Purpose

The model aims to simulate the reproductive dynamics of *D. suzukii* females, which can mate multiple times and store sperm in a spermatheca, i.e. a specialized organ in female that stores sperm after mating. Different sperm biases are tested to evaluate their influence on population dynamics when sterilized males are released. Although not the primary objective of this study, the model also serves as a framework to explore release scenarios, by assessing how different release rates affect population dynamics.

S5.1.2 Entities, State Variables, and Scales

The model consists of four types of agents: sterilized males S, wild males M, larvae L (including all immature sub-stages: eggs, larvae and pupae) and females F.

The model operates on discrete time steps, tracking population dynamics over multiple days, and is spatially implicit.

S5.1.3 Process Overview and Scheduling

At each time step, the time counter is incremented. When sterilized males are scheduled for release, they are added to the population. Subsequently, agents execute their category-specific behaviors, such as mortality, mating, and egg-laying. Agents are processed randomly at each time step to prevent ordering biases. Finally, data collection is performed to record population statistics and agent attributes. Figure 3 illustrates the process for a given time step for each agent type.

S5.2 Design Concepts

S5.2.1 Interactions

In our model, available females can mate at any time, as one of our key assumptions is that males are not a limiting factor. The model is not spatially explicit, meaning that individuals are well-mixed, without any spatial structure. As a consequence, mating events are assumed to be random and depend solely on the proportion of wild and sterilized males in the population.

S5.2.2 Sensing

The model is based on biological principles governing *D. suzukii* reproduction, particularly multiple mating and sperm bias. It incorporates different levels of sperm sensing, which influence how females use stored sperm for fertilization. The level of sensing determines the sperm use biases studied as follows:

- **No sensing** (*Mixed*): Females do not distinguish between sperm sources.

- ***Mixed***: Egg-laying is proportional to the fertile sperm content of the spermatheca. A scenario in which only a single sperm from a single ejaculate was chosen and used in the spermatheca was tested, and the results of this scenario nearly overlapped with those of the *Mixed* scenario.

- **Limited sensing** (*First, Last*): Females exhibit a simple rule-based selection.
  - ***First***: Females use sperm from their first mating.
  - ***Last***: Females use sperm from their last mating.

- **Strong sensing** (*Preference*): Females actively favor specific sperm types.
  - ***Preference***: Total fertile sperm preference (***Preference W***), intermediate fertile sperm preference (***Preference I***), or total sterile sperm preference (***Preference S***).

S5.2.3 Stochasticity

Key life history traits (e.g., lifespan, mating intervals, emergence times) are modeled using uniform and Poisson distributions with parameters based on published data.

S5.2.4 Observation

Data collection tracks population size by agent type, female mating history and offspring production.

S5.3 Details

S5.3.1 Initialization

The model is initialized with user-defined population sizes, number of sterilized males released and sperm use biases (*First*, *Last*, *Mixed*, *Preference W*, *Preference I* or *Preference S*).

The initialization phase also includes the setup of counters for tracking the population of each agent category, a time counter, and a release counter. A scheduler is created to manage the activation of agents, ensuring that all agents are activated in a random order at each time step. Subsequently, the initial population of agents is created, and a data collector is configured to record global metrics (e.g., total population, number of agents in each category, number of releases).

S5.3.2 Input Data

The model does not require external datasets but relies on empirical parameter values from published studies. All parameters are listed in Table A in S1 Text.

S5.3.3 Initialization of New Agents

Each agent is characterized by a unique identifier (a number), an attribute of category (S, W, L, or F), its creation date (`creation_date`) (i.e., the time step t at which the agent is created in the model), and its death date (`death_date`).

Adult agents (S, W, and F) are assigned a death date as follows:

- If the agent (S, W, or F) is created at time $t = 0$, its lifespan is drawn from a uniform distribution between minimum and maximum lifespan found in the literature, an initial age is randomly assigned between 0 and its lifespan, and its death date is adjusted accordingly based on the lifespan and initial age.

- If the agent (S, W, or F) is created at time $t > 0$, its lifespan is drawn from a uniform distribution between minimum and maximum lifespan found in the literature, and its death date is determined at creation by adding this lifespan to the creation date.

The emergence date of larvae (L) is calculated by adding a randomly drawn development duration to its creation date. However, it will only emerge if it survives all sub-stages. A mortality probability, depending on the sub-stage (eggs, larvae, pupae), is applied at each time step (see Table ??).

Females (F) have additional characteristics: their spermatheca, the total number of eggs laid (`egg_laid`) and the time remaining until their next mating (`time_until_next_mating`). The number of eggs laid and the time until next mating are initialized to zero. The spermatheca content corresponds to the number of matings with wild males (`fertile_matings`) and sterilized males (`sterile_matings`), as well as the details of their first (`first_mating`) and last mating (`last_mating`) events. The former are initialized to 0, the latter to "None". New females are hence considered to be virgin and ready to mate.

Upon creation, the counter for the agent category in the model is incremented. The initially created agents perform their steps at time 0, so the structure of the female population (fertilized vs. infertile) depends on the structure of the male population (number of wild and sterilized males at the initial time).

S5.3.4 Submodels

At each time step t,

Sterilized Male Release New sterilized males are added to the population, simulating the release of sterilized males as part of SIT.

Mortality At each time step, the survival status of all agents is evaluated by comparing the current time to their death date. If $t \geq$ `death_date`, the agent is removed from the scheduler, and the corresponding category counter is decremented.

Emergence Larvae undergo an additional check: if $t \geq$ `emergence_date`, they transition to adulthood, becoming either a wild male or a female according to a Bernoulli distribution with a 0.5 probability for each sex. A new agent (male or female) is then created and integrated into the model.

Mating Females assess their availability for mating based on the value of `time_until_next_mating`. If they are available (`time_until_next_mating` $= 0$), they mate with either a wild or a sterilized male, depending on the proportion of each male type in the population. The spermatheca is updated according to the mating outcome. If both `fertile_matings` and `sterile_matings` are 0, then `first_mating` is set to 1 if the mating is with a wild male, and 0 otherwise. If either `fertile_matings` or `sterile_matings` is greater than 0, then `last_mating` is set to 1 if the mating is with a wild male, and 0 otherwise. The value of `first_matings` or `sterile_matings` is increased by 1, depending of the male the female mated with. The time until the next mating is updated to reflect the refractory rate associated with the male the female mated with. If the female is not available (`time_until_next_mating` > 0), then `time_until_next_mating` is decremented by 1.

Sperm Use Upon mating, sperm is used according to the selected sperm use bias (`sperm_selection`), as described in section S5.2.2. The use of sperm refers to testing whether fertile sperm is used or not, depending on the scenario being explored.

- In the *First* scenario, the first mating determines the sperm use,
- whereas in the *Last* scenario, it is the last mating.
- In the *Mixed* scenario, egg-laying is proportional to the amount of fertile sperm stored.
- In the "Preference" scenarios, when there is a complete preference for fertile (*Preference W*) or sterile sperm (*Preference S*), we simply check if the prefered sperm is stored, and if so, egg-laying will occur or not. When the preference is intermediate (*Preference I*), the preference rate decreases the probability of using fertile sperm to fertilize the eggs.

Egg Laying During the egg-laying step, females that have stored fertile sperm (`fertile_matings` > 0) may use it to fertilize eggs. The number of eggs laid (`egg_laid`) per time step is drawn from a Poisson distribution, where the mean depends on a logistic growth function that limits larval production as density approaches the carrying capacity. The egg-laying behavior depends on the implemented sperm use bias. In the *First* and *Last* scenarios, females lay eggs only if the sperm from their first (`first_mating` $== 1$) or last (`last_mating` $== 1$) mating, respectively, came from a fertile male. In contrast, in the *Mixed* scenario, the number of eggs laid is proportional to the proportion of fertile sperm stored, computed as the ratio between fertile and total mating. This proportion is then applied to scale the expected number of eggs. This allows for partial fertility when both sterile and fertile sperm are present. The scenarios of preference incorporate probabilistic egg-laying decisions based on the relative amounts of fertile and sterile sperm stored, modulated by a preference parameter $\widetilde{\eta}$. When $0 < \widetilde{\eta} < 1$, corresponding to the *Preference I* scenario, the probability of using fertile sperm is given by:

$$P = \frac{\texttt{fertile\_matings}}{\texttt{fertile\_matings} + \widetilde{\eta} \times \texttt{sterile\_matings}}$$

When $\widetilde{\eta} = 0$ (*Preference W*), females exclusively use fertile sperm when available. Conversely, when $\widetilde{\eta} = 1$ (*Preference S*), females use sterile sperm by default if available; otherwise, if only fertile sperm is stored, they proceed to lay eggs, with the number of eggs drawn from a Poisson distribution as previously described.

In all cases, each fertilized egg results in the creation of a new larval agent, which is added to the simulation.
