# Supplementary material for: Polyandry: A threat or an opportunity for the sterile insect technique?
Source: PLoS Comput Biol. 2026 Apr 29;22(4):e1014212. doi: 10.1371/journal.pcbi.1014212 (PMC13143183; doi:10.1371/journal.pcbi.1014212)
Supplement: S6 Text — (PDF) [file pcbi.1014212.s006.pdf]

S6 Comparison Between the Compartmental Model and the Agent-Based Model

This appendix compares the larval dynamics obtained from the compartmental model (Eq. (Eq. 2), Fig. B (A-B)) and the agent-based model (ABM) (Sect. 2.2, Fig. B (C-D)) across different sterilized male release rates (σ) and sperm use scenarios (described in Sect. 3.1.1). The goal is to evaluate the consistency between the two approaches and to highlight both their convergences and divergences in simulating larval population dynamics. The simulated release rates σ in the two subsequent scenarios (high infestation and low infestation) differ substantially, as they are adjusted to the initial conditions, to illustrate different contexts such as population suppression or maintenance.

High infestation The simulations, presented in Fig. A, are conducted under the same initial conditions as in Fig. 4A, allowing for direct comparison. To start with only fertilized females (F_F in the compartmental model) in the ABM, the females present in the system at the initial time have fertile sperm stored in their spermatheca. As observed with the compartmental model, when release rates are below the eradication thresholds for the *First* and *Last* scenarios, larval suppression is initially more rapid and pronounced in the *Last* scenario (which includes multiple mating). However, over the long term, this suppression proves less effective than in the *First* scenario, thus confirming the reversal in trend already noted.

When the release rate is sufficiently high, the *Last* scenario still leads to a faster suppression of the larval population, although eradication is achieved in all cases. The intermediate case observed in the compartmental model, where the population is maintained only in the *Last* scenario for intermediate σ, is not reproduced with the agent-based model.

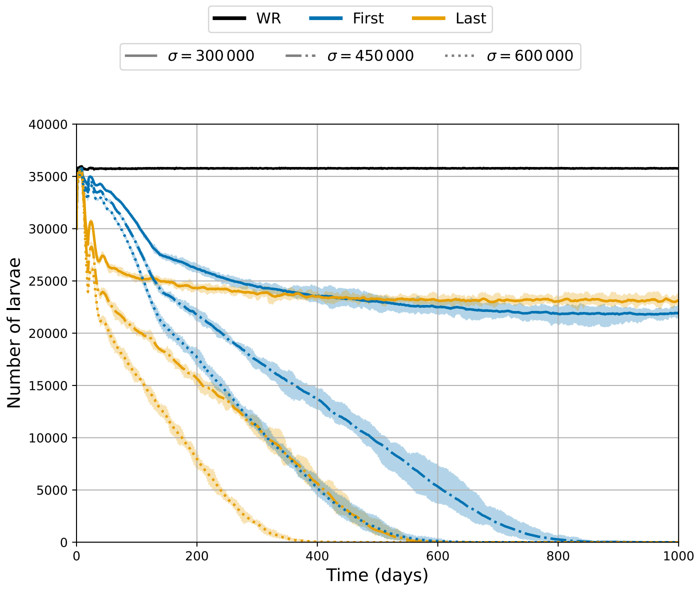

Fig A. Simulations of larval density L over time for different initial infestation levels and sterilized male release rates (σ). This figure shows results for a high initial infestation level (initial conditions: $L = 30{,}000$, $M = 100{,}000$, $F_I = 0$, $F_F = 100{,}000$, $S = \frac{\sigma}{\mu_S}$). Black curves represent the scenario without releases ($\sigma = 0$). Blue curves show the *First* sperm usage scenario ($\tau_I = \tau_F = 0$), and orange curves show the *Last* scenario ($\tau_I > 0$, $\tau_F > 0$). Solid curves correspond to $\sigma = 300{,}000$ (below the eradication threshold in Fig. 2), dash-dot curves to $\sigma = 450{,}000$ (between thresholds), and dotted curves to $\sigma = 600{,}000$ (above the eradication threshold). Each curve represents the mean of 10 stochastic simulations, and the shaded area around each line shows the range between the minimum and maximum larval densities across replicates.

Low infestation Under the "without release" scenario (*WR*, Fig. B), both models yield similar larval dynamics, with populations gradually stabilizing around the carrying capacity K. However, the ABM displays slightly slower convergence.

For varying release rates tested in Fig. B, the overall trends and curve shapes remain consistent across models. The compartmental model generally reaches equilibrium about twice as quickly. At $\sigma = 10{,}000$, the compartmental model shows faster convergence in the *Last* scenario compared to the *First*, while this distinction is not observed in the agent-based model.

At higher release rates ($\sigma = 25{,}000$), the divergence becomes more evident. In the compartmental model, the larval population still tends to stabilize near K for both sperm usage scenarios. In contrast, in the agent-based model, the population stabilizes much more slowly in the *First* scenario, and it fails to persist altogether in the *Last* scenario. Consequently, the relative reductions in larval populations differ considerably between the two approaches.

At very high release rates ($\sigma = 50{,}000$), neither model maintains the larval population, regardless of the sperm usage scenario, indicating successful suppression in all cases.

Despite differences in convergence speeds and long-term behavior, both models consistently rank sperm usage scenarios in the same order: *Last* yields more effective short-term suppression than *First*, as previously discussed in Sect. 2.1. This consistency reinforces the robustness of qualitative insights while showcasing the complementarity of both approaches.

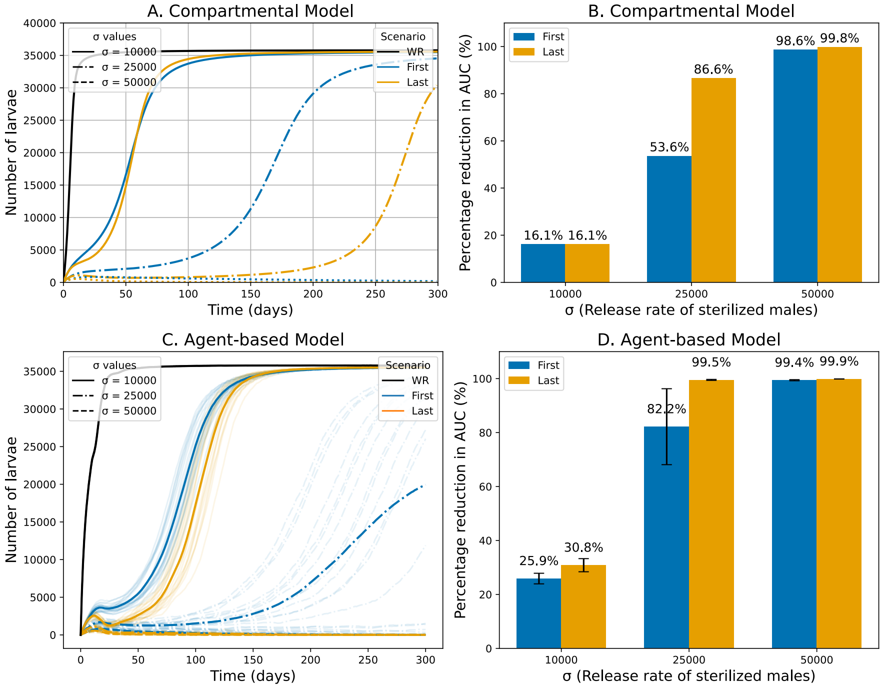

Fig B. Comparison of larval dynamics and percentage reduction in areas under the curves between the compartmental model (A-B) (Eq. 2) and the agent-based model (described in Sect. 2.2, C-D) under different sterilized male release rates (σ) and sperm usage scenarios. Simulations were initialized with 1000 wild males, 1000 females, and 0 larvae. (A-C) Larval density over time. Black curves represent the scenario without sterilized male releases ($\sigma = 0$). Blue curves correspond to the *First* sperm usage scenario, and orange curves to the *Last* scenario. Solid, dash-dot, and dotted lines indicate $\sigma = 10{,}000$, $\sigma = 25{,}000$, and $\sigma = 50{,}000$, respectively. For the agent-based model, results are based on 20 simulations, with the main (thicker) curve representing the average of these simulations. (B-D) Percentage reduction in the area under the curve relative to the reference scenario (*WR*, no release), for the compartmental model (B) and the agent-based model (D).
